# Rosuvastatin Versus Atorvastatin for Cardiovascular Disease Risk in Patients with Type 2 Diabetes: A Korean Cohort Study

**DOI:** 10.3390/ph18121860

**Published:** 2025-12-05

**Authors:** Chaeyoon Kim, Junhyuk Chang, Sujin Gan, Sohyeon Park, Kalynn Park, Hong-Ah Kim, Rae Woong Park, Sandy Jeong Rhie

**Affiliations:** 1 Graduate School of Pharmaceutical Sciences, Ewha Womans University, Seoul 03760, Republic of Korea; miseong@ewhain.net (C.K.); qkrthgus9129@ewha.ac.kr (S.P.); ngpark93@ewha.ac.kr (K.P.); 2 Department of Biomedical Sciences, Graduate School of Ajou University, 206, World cup-ro, Yeongtong-gu, Suwon-si 16499, Republic of Korea; wkd9504@ajou.ac.kr (J.C.); gansujin@gmail.com (S.G.); veritas@ajou.ac.kr (R.W.P.); 3 College of Pharmacy, Kyung Hee University, 26 Kyungheedae-ro, Dongdaemun-gu, Seoul 02453, Republic of Korea; 4 Department of Biomedical Informatics, Ajou University School of Medicine, 206, World cup-ro, Yeongtong-gu, Suwon-si 16499, Republic of Korea

**Keywords:** cardiovascular events, elderly, peripheral artery disease, statin, type 2 diabetes

## Abstract

**Background**: Rosuvastatin and atorvastatin are indicated for cardiovascular protection in patients with type 2 diabetes (T2D) but may differ in clinical effectiveness and safety. This study compared real-world cardiovascular and safety outcomes associated with rosuvastatin versus atorvastatin in patients with T2D, with an emphasis on older adults. **Methods**: This retrospective cohort study used electronic health records from 10 Korean hospitals standardized to the Common Data Model. Adults (≥18 years) with T2D who were newly prescribed rosuvastatin or atorvastatin were included. After propensity score matching, primary outcomes (myocardial infarction [MI], heart failure, stroke, cardiac arrest, and in-hospital death), secondary outcomes (peripheral arterial disease [PAD] and glaucoma), and safety outcomes (acute kidney injury, cataract, and myalgia) were compared. Subgroup and sensitivity analyses were conducted among patients aged ≥65 years. **Results**: Among 49,034 patients (rosuvastatin, 16,123; atorvastatin, 32,911), baseline characteristics were well balanced. Across all participating hospitals, the comparative analyses showed no meaningful differences in cardiovascular or safety outcomes between the two statins. However, among patients aged ≥65 years, rosuvastatin was associated with a higher risk of PAD (hazard ratio [HR] 1.19; 95% confidence interval [CI] 1.03–1.38), and this finding was consistent in sensitivity analysis (HR 1.31; 95% CI 1.01–1.70). **Conclusions**: Rosuvastatin and atorvastatin demonstrated comparable cardiovascular effectiveness and safety. Although rosuvastatin was associated with a modestly higher incidence of PAD in older adults, the difference was small. From a clinical perspective, these findings underscore the importance of individualized statin therapy tailored to patient-specific factors such as age, comorbidity, and vascular health. Overall, both statins provide overall therapeutic equivalence for cardiovascular prevention.

## 1. Introduction

Diabetes is a major risk factor for cardiovascular disease (CVD), a leading cause of mortality worldwide. In 2021, CVD accounted for approximately 19.91 million deaths globally [1]. Type 2 diabetes (T2D), the most prevalent form of diabetes, is characterized by insulin resistance and progressive β-cell dysfunction, leading to chronic hyperglycemia and multisystem complications [2]. A key pathophysiological link between T2D and CVD is diabetic dyslipidemia, a pro-atherogenic lipid profile defined by elevated levels of triglycerides, free fatty acids, and small dense low-density lipoprotein cholesterol (LDL-C), combined with reduced high-density lipoprotein cholesterol (HDL-C) [3]. Insulin resistance and increased hepatic lipogenesis further exacerbate this atherogenic state, accelerating the progression of atherosclerosis.

Statins are recommended as the first-line lipid-lowering therapy for patients with T2D [4,5]. As HMG-CoA reductase inhibitors, statins exert their primary therapeutic effect by inhibiting hepatic cholesterol synthesis, which upregulates LDL receptors and enhances the clearance of circulating LDL-C. They also reduce the assembly and secretion of very-low-density lipoprotein (VLDL), further lowering LDL-C levels [6]. Beyond lipid-lowering, statins exhibit anti-inflammatory, antioxidant, antithrombotic, and plaque-stabilizing actions by reducing markers such as high-sensitivity C-reactive protein, prothrombin fragment 1+2, soluble tissue factor, and von Willebrand factor antigen [7]. In South Korea, rosuvastatin and atorvastatin account for approximately 83% of all statin prescriptions, reflecting their widespread use in clinical practice [8].

Clinical trial data suggest that rosuvastatin may provide greater lipid-modifying potency; however, this does not always translate into superior clinical outcomes, and may be associated with an increased risk of adverse effects, such as myopathy, proteinuria, and rarely, acute kidney injury (AKI), particularly in older adults [9]. Additionally, older adults remain underrepresented in statin trials, and their physiological vulnerabilities, including altered drug metabolism and higher rates of polypharmacy, raise concerns regarding tolerability and safety [10,11]. Notably, head-to-head, real-world comparisons of rosuvastatin versus atorvastatin in older adults with T2D are scarce. Most studies either aggregate age strata [12,13,14,15], focus on lipid surrogates rather than hard outcomes [16], or insufficiently account for routine-care dynamics, such as dose intensity, treatment switching, and concomitant lipid-lowering therapy. This evidence gap has substantial clinical and assistive implications, as it limits individualized statin selection. Therefore, age-specific, real-world, comparative-effectiveness data that prioritize clinical outcomes and safety are needed.

As statin therapy in diabetes is a cornerstone of cardiovascular prevention, understanding the comparative real-world performance of different statins is critical for optimizing patient-centered treatment strategies, particularly in aging populations. Evidence from such studies can directly inform clinical decision-making, enhance drug safety monitoring, and support precision pharmacotherapy in diabetes care. The primary objective of this study was to compare the risk of a composite of major cardiovascular events (myocardial infarction [MI], heart failure, stroke, cardiac arrest, and in-hospital death) between new users of rosuvastatin and atorvastatin in patients with T2D. Secondary objectives included comparing the risks of peripheral arterial disease (PAD) and glaucoma, as well as key safety outcomes, including AKI, cataract, and myalgia.

## 2. Results

### 2.1. Cohort Composition, Lipid Control, and Statin Dose Intensity

**Figure 1:**
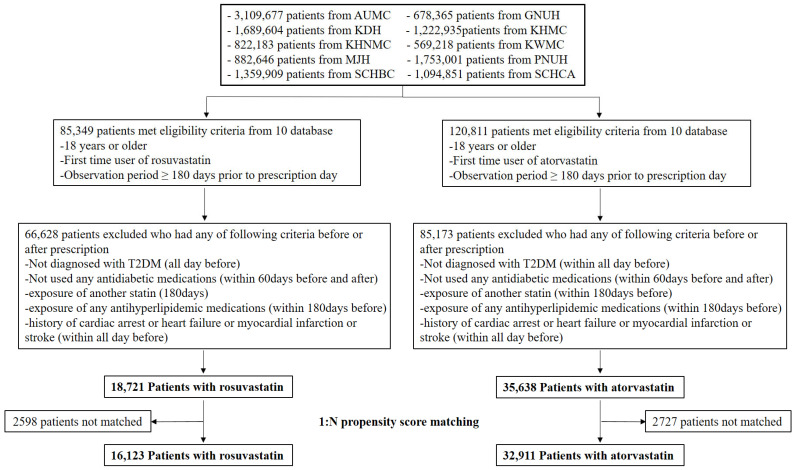
Study flowchart of patients receiving rosuvastatin vs. atorvastatin. Abbreviations: AUMC, Ajou University Medical Center; GNUH, Gyeongsang National University Hospital; KDH, Kangdong Sacred Heart Hospital; KWMC, Kangwon National University Hospital; KHMC, Kyunghee Medical Center; KHNMC, Kyunghee University Hospital at Gangdong; MJH, Myongji Hospital; PNUH, Pusan National University Hospital; SCHBC, Soonchunhyang University Hospital at Bucheon; SCHCA, Soonchunhyang University Hospital at Cheonan; T2D, Type 2 diabetes.

**Figure 2:**
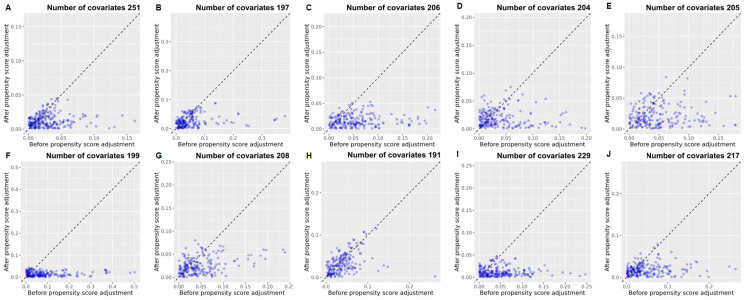
Covariate balance plot before and after PSM across 10 hospitals. (**A**) AUMC; (**B**) GNUH; (**C**) KDH; (**D**) KHMC; (**E**) KHNMC; (**F**) KWMC; (**G**) MJH; (**H**) PNUH; (**I**) SCHBC; (**J**) SCHCA. Abbreviations: AUMC, Ajou University Medical Center; GNUH, Gyeongsang National University Hospital; KDH, Kangdong Sacred Heart Hospital; KWMC, Kangwon National University Hospital; KHMC, Kyunghee Medical Center; KHNMC, Kyunghee University Hospital at Gangdong; MJH, Myongji Hospital; PNUH, Pusan National University Hospital; SCHBC, Soonchunhyang University Hospital at Bucheon; SCHCA, Soonchunhyang University Hospital at Cheonan.

Adult patients (aged ≥18 years) diagnosed with T2D and newly prescribed rosuvastatin or atorvastatin were identified across 10 tertiary hospitals in South Korea. The databases were de-identified and standardized to the Observational Health Data Sciences and Informatics Common Data Model (OMOP-CDM, version 5.3.1) [17,18]. Baseline differences were addressed using large-scale propensity score matching (PSM) with regularized logistic regression across a comprehensive array of demographic, clinical, and laboratory covariates. Primary analyses employed an intention-to-treat (ITT) framework, in which patients were analyzed according to their initial statin prescription at the index date, regardless of subsequent discontinuation, switching, or addition of lipid-lowering therapy. Sensitivity analyses utilized an as-treated (AT) approach, restricting follow-up to continuous exposure to the initial statin and censoring at the time of treatment change. After 1:N PSM, a total of 49,034 patients from the 10 databases were included, comprising 16,123 new users of rosuvastatin and 32,911 new users of atorvastatin (Figure 1). Mean follow-up was 4.3 years in the rosuvastatin group and 4.8 years in the atorvastatin group. Across the 10 hospital databases, PSM successfully balanced a large number of baseline covariates, ranging from 191 at PNUH to 251 at AUMC (Figure 2). As demonstrated in Figure 2, most standardized mean differences were below 0.1 after matching, indicating excellent comparability between the treatment groups.

Before PSM, analysis of the cohort demonstrated that the proportion of patients achieving LDL-C levels <70 mg/dL increased substantially within the first year of treatment. This proportion rose from 12.31% to 39.25% in the rosuvastatin group and from 10.58% to 29.31% in the atorvastatin group, indicating similar overall lipid-lowering trends (Supplementary Materials, Figure S1A,B). This figure also illustrates the lipid control status of pre-PSM patients: bar plots depict the distribution of LDL-C levels (<70 mg/dL vs. ≥70 mg/dL) at each time interval, while the line plot indicates the percentage of the total cohort with an available LDL-C measurement at each time point.

In the dose pathway analysis, 91.7% of patients in the rosuvastatin group and 93.2% in the atorvastatin group received moderate-intensity doses. Most patients in both groups (94.3% in the rosuvastatin group and 90.7% in the atorvastatin group) maintained their prescribed dosage throughout the study period (Supplementary Materials, Figures S2 and S3). These findings indicate that equivalent doses were likely prescribed between the two groups, suggesting that dose intensity is unlikely to have affected the outcomes. Dosage information was available for most patients in the atorvastatin group, whereas data for patients in the rosuvastatin group were available from only eight hospitals.

### 2.2. Baseline Covariate Balance and Multicenter Comparative Outcome Analysis

Table 1 presents the baseline characteristics of patients from one study hospital (SCHBC), both before and after PSM (Table 1). Medications were grouped by class, and within each class, the drug with the highest standardized difference after PSM was selected to represent the group. Before PSM, the rosuvastatin group had a higher proportion of patients receiving calcium channel blockers, nitrates, and other antiplatelet drugs, whereas the atorvastatin group had a greater proportion of patients receiving aspirin. After PSM, baseline covariates were well balanced. Consistent results were observed across all databases from other hospitals (Supplementary Materials, Tables S1–S9).

**Figure 3:**
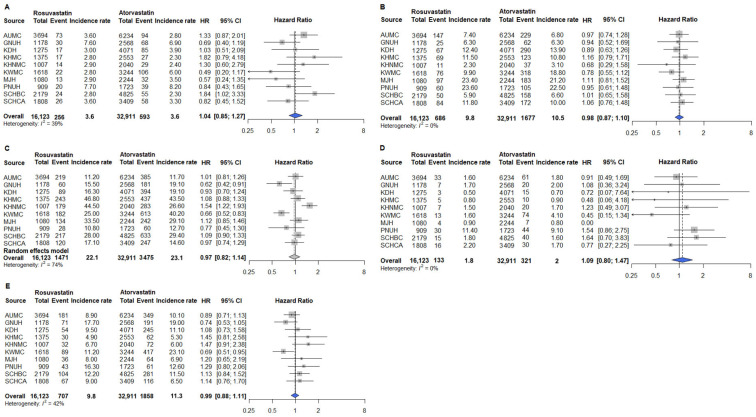
Primary outcomes in cardiovascular event prevention among rosuvastatin and atorvastatin users in the overall population. (**A**) MI; (**B**) Heart failure; (**C**) Stroke; (**D**) Cardiac arrest; (**E**) In-hospital death. Outcomes analyzed using fixed-effect models are represented by a blue diamond, whereas outcomes analyzed with a random-effect model are represented by a gray diamond. Abbreviations: AUMC, Ajou University Medical Center; GNUH, Gyeongsang National University Hospital; KDH, Kangdong Sacred Heart Hospital; KWMC, Kangwon National University Hospital; KHMC, Kyunghee Medical Center; KHNMC, Kyunghee University Hospital at Gangdong; MJH, Myongji Hospital; PNUH, Pusan National University Hospital; SCHBC, Soonchunhyang University Hospital at Bucheon; SCHCA, Soonchunhyang University Hospital at Cheonan.

**Figure 4:**
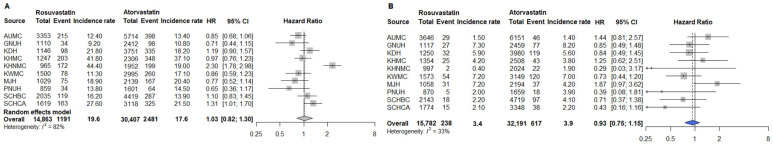
Secondary outcomes among rosuvastatin and atorvastatin users in the overall population. (**A**) PAD; (**B**) Glaucoma. Outcomes analyzed using fixed-effect models are represented by a blue diamond, whereas outcomes analyzed using a random-effect model are represented by a gray diamond. Abbreviations: AUMC, Ajou University Medical Center; GNUH, Gyeongsang National University Hospital; KDH, Kangdong Sacred Heart Hospital; KWMC, Kangwon National University Hospital; KHMC, Kyunghee Medical Center; KHNMC, Kyunghee University Hospital at Gangdong; MJH, Myongji Hospital; PNUH, Pusan National University Hospital; SCHBC, Soonchunhyang University Hospital at Bucheon; SCHCA, Soonchunhyang University Hospital at Cheonan.

**Figure 5:**
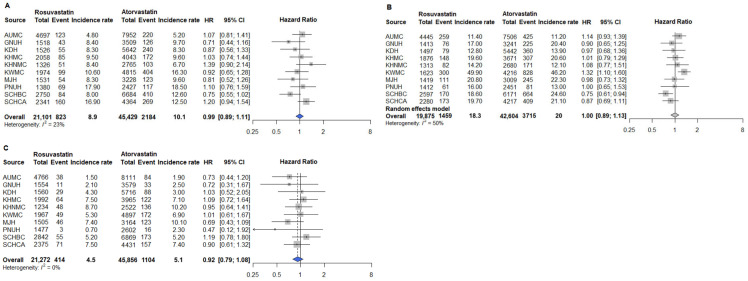
Safety outcomes among rosuvastatin and atorvastatin users in the overall population. (**A**) AKI; (**B**) Cataract; (**C**) Myalgia. Outcomes analyzed using fixed-effect models are represented by a blue diamond, whereas outcomes analyzed using a random-effect model are represented by a gray diamond. Abbreviations: AUMC, Ajou University Medical Center; GNUH, Gyeongsang National University Hospital; KDH, Kangdong Sacred Heart Hospital; KWMC, Kangwon National University Hospital; KHMC, Kyunghee Medical Center; KHNMC, Kyunghee University Hospital at Gangdong; MJH, Myongji Hospital; PNUH, Pusan National University Hospital; SCHBC, Soonchunhyang University Hospital at Bucheon; SCHCA, Soonchunhyang University Hospital at Cheonan.

The primary outcomes included MI, heart failure, stroke, cardiac arrest, and in-hospital death. Secondary outcomes included PAD and glaucoma. Safety outcomes included AKI, cataract, and myalgia. After PSM, analyses conducted across the 10 participating institutions showed no significant differences in the risks of primary and secondary outcomes between the rosuvastatin and atorvastatin groups (Figure 3 and Figure 4). Similarly, safety outcomes did not significantly differ between patients receiving rosuvastatin and those receiving atorvastatin after PSM across the 10 institutions (Figure 5). Collectively, these results indicate that rosuvastatin and atorvastatin provide equivalent cardiovascular protection and safety in adults with T2D in real-world, multicenter settings.

### 2.3. Sensitivity Analyses

Analysis of primary outcomes using the AT approach revealed no significant differences between the rosuvastatin and atorvastatin groups across the participating institutions (Supplementary Materials, Figure S4). Similarly, for secondary and safety outcomes, no differences were observed between the rosuvastatin and atorvastatin groups when analyzed using the AT approach (Supplementary Materials, Figures S5 and S6).

### 2.4. Subgroup Analysis in Patients Aged ≥65 Years

**Figure 6:**
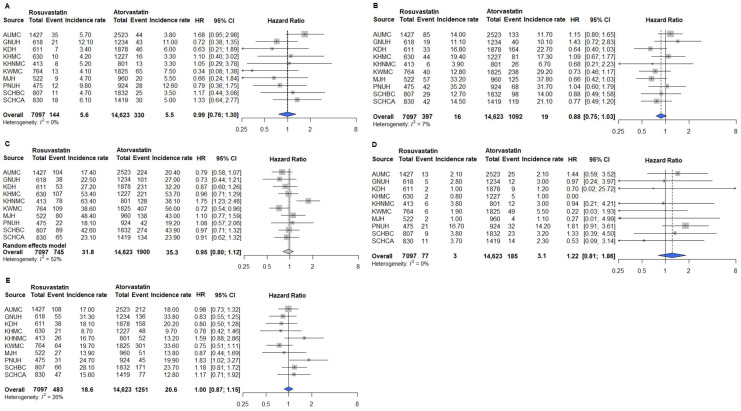
Primary outcomes among rosuvastatin and atorvastatin users aged ≥65 years. (**A**) MI; (**B**) Heart failure; (**C**) Stroke; (**D**) Cardiac arrest; (**E**) In-hospital death. Outcomes analyzed using fixed-effect models are represented by a blue diamond, whereas outcomes analyzed with a random-effect model are represented by a gray diamond. Abbreviations: AUMC, Ajou University Medical Center; GNUH, Gyeongsang National University Hospital; KDH, Kangdong Sacred Heart Hospital; KWMC, Kangwon National University Hospital; KHMC, Kyunghee Medical Center; KHNMC, Kyunghee University Hospital at Gangdong; MJH, Myongji Hospital; PNUH, Pusan National University Hospital; SCHBC, Soonchunhyang University Hospital at Bucheon; SCHCA, Soonchunhyang University Hospital at Cheonan.

**Figure 7:**
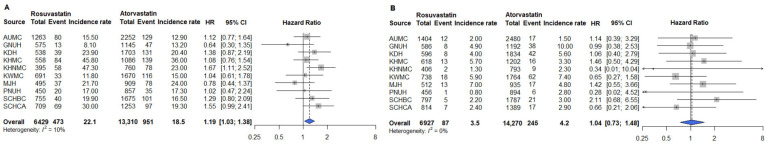
Secondary outcomes among rosuvastatin and atorvastatin users aged ≥65 years. (**A**) PAD; (**B**) Glaucoma. Abbreviations: AUMC, Ajou University Medical Center; GNUH, Gyeongsang National University Hospital; KDH, Kangdong Sacred Heart Hospital; KWMC, Kangwon National University Hospital; KHMC, Kyunghee Medical Center; KHNMC, Kyunghee University Hospital at Gangdong; MJH, Myongji Hospital; PNUH, Pusan National University Hospital; SCHBC, Soonchunhyang University Hospital at Bucheon; SCHCA, Soonchunhyang University Hospital at Cheonan.

**Figure 8:**
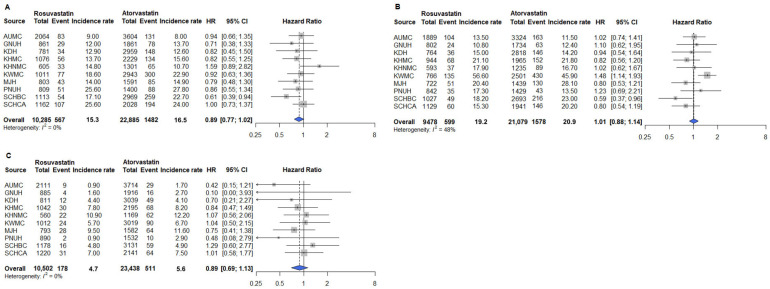
Safety outcomes among rosuvastatin and atorvastatin users aged ≥65 years. (**A**) AKI; (**B**) Cataract; (**C**) Myalgia. Abbreviations: AUMC, Ajou University Medical Center; GNUH, Gyeongsang National University Hospital; KDH, Kangdong Sacred Heart Hospital; KWMC, Kangwon National University Hospital; KHMC, Kyunghee Medical Center; KHNMC, Kyunghee University Hospital at Gangdong; MJH, Myongji Hospital; PNUH, Pusan National University Hospital; SCHBC, Soonchunhyang University Hospital at Bucheon; SCHCA, Soonchunhyang University Hospital at Cheonan.

**Figure 9:**
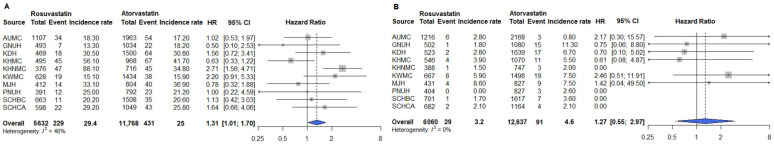
Secondary outcomes among rosuvastatin and atorvastatin users aged ≥65 years. (**A**) PAD; (**B**) Glaucoma. Abbreviations: AUMC. Ajou University Medical Center; GNUH, Gyeongsang National University Hospital; KDH, Kangdong Sacred Heart Hospital; KWMC, Kangwon National University Hospital; KHMC, Kyunghee Medical Center; KHNMC, Kyunghee University Hospital at Gangdong; MJH, Myongji Hospital; PNUH, Pusan National University Hospital; SCHBC, Soonchunhyang University Hospital at Bucheon; SCHCA, Soonchunhyang University Hospital at Cheonan.

Subgroup analyses were conducted to assess whether outcomes differed in patients aged ≥65 years compared with the overall population. After 1:N PSM, pooled data included 7097 patients receiving rosuvastatin and 14,623 receiving atorvastatin. Across all institutions, no significant differences were observed in the risks of primary cardiovascular outcomes, including MI, heart failure, stroke, cardiac arrest, and in-hospital death between rosuvastatin and atorvastatin (Figure 6). Pooled hazard ratios [HRs] were close to unity, and the confidence intervals (CIs) were narrow, indicating that both statins provided equivalent overall cardiovascular protection in this population. A divergence was observed for PAD, one of the secondary outcomes. In the ITT analysis, rosuvastatin users had a 19% higher risk of PAD compared with atorvastatin users (HR 1.19; 95% CI 1.03–1.38). In contrast, glaucoma (Figure 7B) and the three safety outcomes—AKI, cataract, and myalgia (Figure 8)—showed no difference between groups, with HRs close to 1.0. These findings were consistent in the sensitivity analyses using the AT approach. Risks for primary outcomes (Supplementary Materials, Figure S7) and safety outcomes (Supplementary Materials, Figure S8) remained neutral, confirming the robustness of the main results. However, for secondary outcomes, the association between rosuvastatin and PAD persisted in the AT analysis (HR 1.31; 95% CI 1.01–1.70; Figure 9A), indicating that the signal was not attributable to treatment discontinuation or switching. Other secondary outcomes, such as glaucoma (Figure 9B), again showed no meaningful difference.

**Figure 10:**
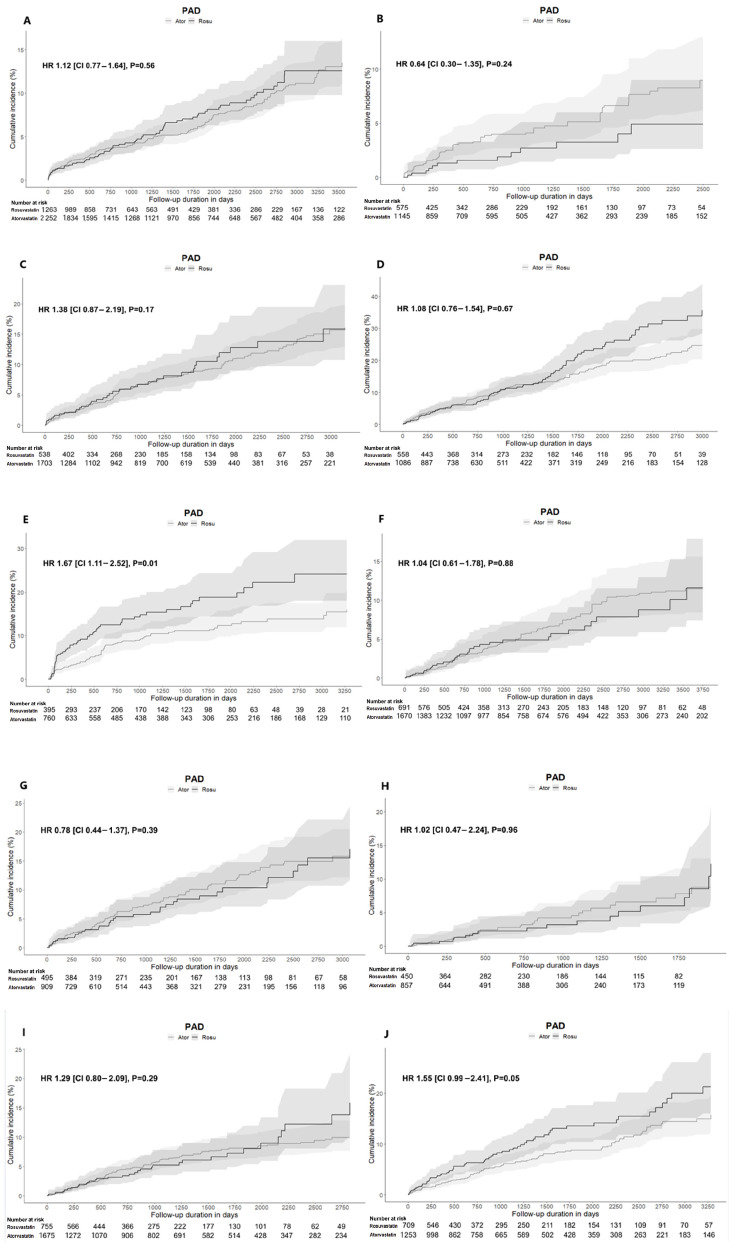
Kaplan–Meier plots comparing the risk of PAD among rosuvastatin and atorvastatin users aged ≥65 years across 10 hospitals after PSM. (**A**) AUMC; (**B**) GNUH; (**C**) KDH; (**D**) KHMC; (**E**) KHNMC; (**F**) KWMC; (**G**) MJH; (**H**) PNUH; (**I**) SCHBC; (**J**) SCHCA. Abbreviations: AUMC, Ajou University Medical Center; GNUH, Gyeongsang National University Hospital; KDH, Kangdong Sacred Heart Hospital; KWMC, Kangwon National University Hospital; KHMC, Kyunghee Medical Center; KHNMC, Kyunghee University Hospital at Gangdong; MJH, Myongji Hospital; PNUH, Pusan National University Hospital; SCHBC, Soonchunhyang University Hospital at Bucheon; SCHCA, Soonchunhyang University Hospital at Cheonan.

A Kaplan–Meier analysis was performed to illustrate the cumulative incidence of PAD over time. Figure 10 presents the cumulative incidence curves for patients aged ≥65 years across all 10 hospitals. While the curves overlapped in most hospitals, the patterns observed at KHNMC (Figure 10E) were consistent with the overall trends shown across institutions (Figure 7A and Figure 9A). At this institution, the cumulative incidence curve for the rosuvastatin group (solid line) gradually diverged from that of the atorvastatin group (dashed line), consistently remaining higher over time. This gap became progressively more pronounced, indicating that the statistically observed risk increase identified in the HR analysis translates into a measurable difference in cumulative incidence over time.

## 3. Discussion

### 3.1. Comparative Effectiveness of Rosuvastatin and Atorvastatin

In patients with T2D, rosuvastatin and atorvastatin demonstrated comparable effectiveness and safety across a range of cardiovascular and systemic outcomes, including MI, heart failure, stroke, cardiac arrest, in-hospital death, PAD, glaucoma, AKI, cataract, and myalgia. Despite rosuvastatin’s well-documented potency in improving LDL-C and HDL-C levels [19,20], no significant differences were observed in major cardiovascular outcomes between rosuvastatin and atorvastatin. These findings are consistent with previous meta-analyses in cardiovascular populations [21].

To minimize potential confounding related to statin intensity, prescribing patterns were analyzed, confirming that both groups predominantly received moderate-intensity statin regimens and achieved similar reductions in LDL-C to below 70 mg/dL. PSM further ensured balanced baseline cardiovascular risk factors, indicating that any observed outcome differences likely reflect intrinsic pharmacological properties rather than dosage differences.

These findings align with previous research in patients with coronary artery disease, where greater LDL-C reduction with rosuvastatin did not translate into improved clinical outcomes [15]. Werida et al. demonstrated that, in patients with T2D and dyslipidemia, rosuvastatin 10 mg produced significantly greater reductions in HbA1c, LDL-C, triglycerides, total cholesterol, atherogenic index, and inflammatory markers, including high-sensitivity C-reactive protein, sortilin, leptin, and adiponectin compared with atorvastatin 20 mg [22]. However, these benefits did not correspond to improved clinical outcomes, highlighting that superior lipid and metabolic effects may not necessarily confer greater clinical benefit.

In contrast, Zhou et al. [23] reported that rosuvastatin significantly reduced major adverse cardiovascular events (MACE) and all-cause mortality compared with atorvastatin. These differences may reflect variation in cohort composition and baseline comorbidities, whereas the present study primarily included statin-naïve patients with T2D and no prior history of CVD, representing a primary prevention cohort in real-world clinical practice.

### 3.2. PAD Risk in Patients Aged ≥65 Years

Subgroup analysis revealed a significant difference in the risk of PAD among patients aged ≥65 years, with rosuvastatin associated with a higher incidence of PAD than atorvastatin. Although noteworthy, the magnitude of this difference was modest, and its clinical relevance remains uncertain. Given the pharmacologic similarities of the two agents, this finding should not be interpreted as evidence of clinical inferiority. Nevertheless, potential mechanisms may involve differences in physicochemical and pharmacological properties, particularly regarding endothelial function, oxidative stress, and atherosclerotic plaque stability.

Atorvastatin’s active metabolites (ATMs) have been shown to robustly inhibit the oxidation of small dense LDL (sdLDL) with 1.5-fold greater inhibition than large LDL and 4.7-fold greater inhibition than very-low-density lipoprotein (VLDL) [24]. Given the critical role of sdLDL in atherosclerosis, atorvastatin’s capacity to reduce sdLDL oxidation may contribute to preventing PAD progression in older adults with diabetes and vulnerable vascular structure [25,26].

The lipophilic nature of atorvastatin facilitates passive membrane diffusion, resulting in broader tissue distribution and inhibition of NADPH oxidase activity beyond the liver, thereby reducing intracellular reactive oxygen species (ROS) levels and enhancing endothelial resilience. Together, these mechanisms may support stabilization of atherosclerotic plaques and potentially lower PAD risk, particularly in older adults with diabetes [27].

Clinical evidence supports this mechanistic distinction. In a randomized, double-blind study of 36 patients with hyperlipidemia and metabolic syndrome or diabetes, participants received either 10 mg/day of lipophilic atorvastatin or 80 mg/day of hydrophilic pravastatin for 12 weeks. Although both statins similarly lowered LDL-C, only atorvastatin significantly reduced key markers of lipid peroxidation (plasma thiobarbituric acid reactive substances and reactive oxygen metabolites). Endothelial function improved in both groups, with no significant differences between the groups. These findings suggest that lipophilic statins exert more potent antioxidant effects at equipotent LDL-lowering doses compared with hydrophilic statins [28].

Collectively, these findings and prior evidence indicate that lipophilic statins, such as atorvastatin, may confer additional protection against PAD through enhanced antioxidant effects on atherogenic lipoproteins and broader tissue penetration. In contrast, hydrophilic statins, such as rosuvastatin, retain potent hepatic LDL-lowering capabilities but may offer comparatively less direct antioxidant benefit in peripheral arteries [29]. However, based on our results, this potential mechanistic advantage did not translate into a large clinical difference.

PAD prevalence increases with age, reaching approximately 15–20% by 80 years [30]. Older adults are particularly susceptible to cumulative endothelial dysfunction and oxidative stress [31,32], which may amplify differential vascular effects of statin therapy. Diabetes mellitus further elevates PAD risk. Recent large-scale cohort studies report approximately a two-fold higher risk of incident PAD in patients with diabetes, even after multivariable adjustment HR = 1.8–2.0) [33]. Moreover, outcomes among patients with both diabetes mellitus and PAD are markedly worse than among those without diabetes, with up to a fourfold higher risk of limb amputation and a significantly increased risk of all-cause mortality (adjusted HR = 1.12, 95% CI 1.05–1.19) [34,35].

While rosuvastatin’s hydrophilic nature and hepatic selectivity confer strong LDL-C lowering and anti-inflammatory effects, including marked reductions in high-sensitivity C-reactive protein [36,37], its limited extrahepatic penetration may account for the relatively weaker influence on oxidative stress and plaque stability in peripheral vessels.

Sensitivity analyses confirmed the robustness of our findings, demonstrating consistency across both ITT and AT approaches, suggesting that the observed PAD risk differences between the two statins are unlikely to be attributable to random variation or confounding factors. Nevertheless, given the modest absolute effect size, further investigation is warranted to determine its clinical significance.

### 3.3. Clinical and Practical Implications

From a practical perspective, rosuvastatin and atorvastatin provide broadly comparable cardiovascular protection and safety profiles in patients with T2D. Therefore, routine statin selection should be guided by patient-specific considerations, including tolerability, potential drug–drug interactions, renal function, and cost-effectiveness, rather than expectations of differential cardiovascular efficacy.

In patients aged ≥65 years, a modest statistical signal for PAD was observed with rosuvastatin; however, the absolute risk difference was small, limiting its clinical impact. These findings underscore the need for vigilant peripheral vascular assessment and dose optimization in older adults receiving high-intensity statins, rather than avoidance of a particular statin.

Notably, these real-world results align with the 2024 American Diabetes Association and 2023 European Society of Cardiology recommendations, which emphasize individualized statin therapy in diabetes management rather than the superiority of any specific drug [5,38]. Integration of such real-world evidence into clinical decision-support tools may enhance medication review, shared decision-making, and long-term adherence in routine diabetes care.

### 3.4. Strengths and Limitations

This study utilized a large, multicenter, real-world database encompassing multiple regions of Korea. Long follow-up periods—up to 1540 days for rosuvastatin and 1733 days for atorvastatin—allowed for a comprehensive assessment of the long-term effects of statins. Use of the OMOP-CDM framework and a dedicated R package version 4.3.1 for standardized analysis ensured methodological consistency across eight datasets. PSM minimized selection bias, and inclusion of only statin-naïve patients with a 6-month pre-treatment observation period mitigated immortal-time bias [39]. Furthermore, exclusion of patients with previous cardiovascular events at baseline strengthened the focus on primary prevention.

Despite the high CVD risk in patients with T2D, few studies have directly compared clinical outcomes between statins in this population, and even fewer studies have addressed primary prevention outcomes in older adults with diabetes. Given the increasing risk of PAD with age, these findings provide valuable real-world insights to inform treatment guidelines.

This study has some limitations. First, statin dosages were unavailable for all patients in the rosuvastatin group, and serial LDL-C measurements were incomplete across hospitals, limiting evaluation of dose-dependent or age-specific lipid changes during follow-up. Second, although both the ITT and AT approaches were applied, the observational design cannot fully eliminate residual confounding. Third, OMOP-CDM data may be subject to missing information, such as out-of-hospital mortality. Fourth, formal sex-stratified effectiveness and safety analyses were not performed, as the study was not specifically powered to detect treatment-by-sex interactions; potential sex-related heterogeneity cannot be excluded. Fifth, continuity of diabetes management during the study period could not be directly verified. Although it was assumed that patients with T2D maintained ongoing disease management, this could not be confirmed through direct clinical or laboratory data. Finally, the findings are specific to the Korean healthcare system, and generalizability to other populations may be limited.

## 4. Materials and Methods

### 4.1. Data Sources

This multicenter, retrospective cohort study utilized real-world clinical data from 12,421,557 patients across 10 secondary or tertiary hospitals in Korea. All data were standardized to the OMOP-CDM version 5.3.1 and accessed through FeederNet (Federated E-health Big Data for Evidence Renovation Network in Korea, https://feedernet.com, accessed on 15 July 2025), supporting collaboration with the OHDSI (Observational Health Data Sciences and Informatics) networks. The study hospitals and their respective patient populations were: (1) Ajou University Medical Center (AUMC; 2,765,795 patients); (2) Gyeongsang National University Hospital (GNUH; 626,663 patients); (3) Kangdong Sacred Heart Hospital (KDH; 1,240,471 patients); (4) Kangwon National University Hospital (KWMC; 609,300 patients); (5) Kyunghee Medical Center (KHMC; 1,168,640 patients); (6) Kyunghee University Hospital at Gangdong (KHNMC; 920,281 patients); (7) Myongji Hospital (MJH; 882,646 patients); (8) Pusan National University Hospital (PNUH; 1,753,001 patients); (9) Soonchunhyang University Hospital at Bucheon (SCHBC; 1,359,909 patients); (10) Soonchunhyang University Hospital at Cheonan (SCHCA; 1,094,851 patients). Overall, electronic medical records spanned 1986–2023. Hospital-specific data collection periods were: (1) AUMC: 1993/01–2023/11; (2) GNUH: 2009/11–2022/04; (3) KDH: 1986/11–2023/01; (4) KWMC: 2003/01–2023/10; (5) KHMC: 2007/06–2022/02; (6) KHNMC: 2006/01–2023/01; (7) MJH: 2003/09–2022/09; (8) PNUH: 2011/02–2019/08; (9) SCHBC: 2001/02–2023/05; (10) SCHCA: 2003/06–2023/04. Patients were followed until the maximum available data date for each hospital. This study was approved by the Institutional Review Board (IRB) of AUMC (IRB number AJOUIRB-EX-2024-291).

### 4.2. Study Design

This retrospective cohort study was conducted and reported in accordance with the Strengthening the Reporting of Observational Studies in Epidemiology guidelines and its REporting of studies Conducted using Observational Routinely collected health Data extension [40]. Patients aged 18–94 years diagnosed with T2D and newly prescribed rosuvastatin or atorvastatin were included. The index date was defined as the date of first exposure to either rosuvastatin or atorvastatin. Patients were required to have at least 180 days of continuous observation before the index date to reduce bias and minimize confounding from other medications. Inclusion criteria required a prior diagnosis of T2D at any time before the index date, a fasting glucose level ≥ 126 mg/dL, or a hemoglobin A1c level ≥ 6.5%, in combination with a prescription for a hypoglycemic agent (insulin, biguanides, sulfonylureas, alpha-glucosidase inhibitor, thiazolidinedione, meglitinide, dipeptidyl peptidase-4 inhibitor, sodium-glucose cotransporter 2 inhibitor, and glucagon-like peptide-1 receptor agonist) within 30 days before or after the index date.

The exclusion criteria were as follows: (1) prescription of any other statin, including the opposite drug (i.e., rosuvastatin for the atorvastatin group and atorvastatin for the rosuvastatin group), or other statins (simvastatin, pitavastatin, pravastatin, lovastatin, or fluvastatin) within 180 days before the index date; (2) prescription of other cholesterol-lowering medications (ezetimibe, fenofibrate, alirocumab, evolocumab) within 180 days before the index date; (3) history of MACE, including MI, heart failure, stroke, or cardiac arrest, at any time before the index date; (4) history of PAD or glaucoma before the index date (patients excluded for respective outcomes; and (5) for safety outcomes (AKI, cataract, myalgia), patients with a prior history of these conditions at any time before the index date, without excluding those with prior MACE.

### 4.3. Outcomes and Follow-Up

Primary outcomes included MI, heart failure, stroke, cardiac arrest, and in-hospital death. Secondary outcomes included PAD and glaucoma. Safety outcomes included AKI, cataract, and myalgia. Treatment was considered ongoing if a patient received a new prescription for the same drug within 90 days of the most recent prescription. An additional 30 days were added to the end of the persistent exposure period as a surveillance window before cohort exit. During follow-up, censoring events were defined as the initiation of statins (including the alternate study drug) or the use of other cholesterol-lowering medications. Outcomes were assessed starting 30 days after the index date to: (1) exclude events that may have been pre-existing or imminent at baseline (i.e., reverse causality) and unrelated to the new statin exposure; and (2) allow a minimum duration for the pharmacological effects of the statins to manifest. Analyses estimated the effect of assignment to a given treatment regardless of subsequent changes in medication use, in accordance with the ITT approach. In this study, the ITT definition included all outcomes occurring after the index date, even if patients discontinued the initial statin, switched to a different statin, or initiated combination therapy with other lipid-lowering agents. This approach was chosen to reflect real-world treatment dynamics and to preserve the comparability between groups based on their treatment assignment. The ITT approach was contrasted with an AT approach in our sensitivity analysis (detailed in Section 4.6) to assess the effect of continued adherence.

### 4.4. Statistical Analysis

To provide clinical context on lipid management, descriptive analyses of LDL-C levels were conducted within the full pre-matched cohort. Patients with available LDL-C measurements were identified at five distinct time points: within 6 months before the index date, within 1 year after the index date, between 1 and 2 years, between 2 and 3 years, and between 3 and 4 years after the index date. The number of patients in each time period was calculated based on LDL-C levels (<70 mg/dL and ≥70 mg/dL). This repeated cross-sectional analysis did not require the cohort to be identical across time points. Additionally, baseline dose comparability between the two groups was confirmed by analyzing the distribution of prescribed doses at the index date, and subsequent dosing patterns were examined using pathway analysis for patients who switched treatments.

Large-scale PSM was performed to balance the target and comparator groups, minimizing potential confounding from baseline covariate imbalances. Covariates included age, sex, prior conditions (hypertension, dyslipidemia, obesity), medications during the 180 days before study statin exposure (anti-diabetic drugs, anti-hypertensives, thiazide diuretics, other diuretics, nitrates, digoxin, aspirin, other antiplatelet drugs, warfarin, nonsteroidal anti-inflammatory drugs), as well as CHA2DS2-VASc, Diabetes complications severity index, and Charlson Comorbidity Index scores. CHA2DS2-VASc was calculated using congestive heart failure, hypertension, age ≥ 75 years (doubled), diabetes, prior stroke or transient ischemic attack (doubled), or thromboembolism, vascular disease, age 65–74 years, and sex category (female). Propensity scores were estimated using large-scale logistic regression models, and patients were matched using a greedy search method with a caliper of 0.2 times the standard deviation of the propensity score distribution. A 1:N PSM was performed to maximize the number of patients for the rosuvastatin versus atorvastatin comparison. Cox proportional hazards regression analysis was conducted to estimate HRs for outcomes between groups. Kaplan–Meier analysis was used to assess cumulative incidence. A total of 115 falsification endpoints were employed to evaluate systematic error and detect confounding and bias [41,42]. These outcomes, including wrist drop and sarcoidosis, were not expected to differ between the two statins. Incidence rates were calculated per 1000 person-years by dividing the number of events by the total number of person-years at risk. Two-sided *p*-values < 0.05 were considered statistically significant. A post hoc power calculation was conducted to assess whether the study sample size (n = 49,034) was sufficient to detect clinically meaningful differences in cardiovascular outcomes between the two statins. Assuming an event rate of 5% and an HR of 0.90, the study had ˃90% power at a two-sided α = 0.05. Therefore, the observed non-significant differences are unlikely to result from insufficient sample size. All primary statistical analyses, including PSM and Cox proportional hazards regression, were conducted using R (version 4.3.1) and relevant packages such as survival.

### 4.5. Subgroup Analysis

Subgroup analyses were performed to evaluate whether the results differed in patients aged ≥65 years compared with the overall population. All conditions were identical to the primary analysis, except for the age criterion. A 1:N PSM was conducted to compare the effectiveness and safety of rosuvastatin and atorvastatin specifically in older patients.

### 4.6. Sensitivity Analysis

For the sensitivity analysis, an AT approach was adopted, restricting the observation period to the duration during which each patient was actively receiving the initially assigned statin. Outcomes were counted only if they occurred during continuous exposure to the initial treatment. Patients were censored at the time of treatment discontinuation, switching to a different statin, or initiation of other lipid-lowering therapies. This approach enabled assessment of the effect of continued adherence to the assigned medication, in contrast to the broader exposure window employed in the ITT approach.

## 5. Conclusions

In this large real-world cohort of patients with T2D, rosuvastatin and atorvastatin demonstrated broadly comparable effectiveness and safety in preventing major cardiovascular events. Although a statistically significant difference in PAD risk was observed in older adults, favoring atorvastatin, the absolute difference was modest. These findings suggest that, while rosuvastatin may be associated with a slightly higher PAD risk in older adults, both statins provide overall equivalent cardiovascular protection and safety profiles.

From a clinical perspective, these results underscore the importance of individualized statin therapy tailored to patient-specific factors, including age, comorbidity burden, vascular health, and potential for drug–drug interactions, rather than the expectation of substantial differences in efficacy between agents. Further longitudinal and mechanistic studies are warranted to validate these observations and to determine whether lipophilic statins provide incremental protection against peripheral vascular complications in older adults with diabetes.

## Figures and Tables

**Table 1:** Baseline characteristics of patients receiving rosuvastatin vs. atorvastatin in the SCHBC cohort.

	Before PSM Adjustment			After PSM Adjustment		
	Rosuvastatin (n = 2532)	Atorvastatin (n = 5282)	Std. Diff	Rosuvastatin (n = 2187)	Atorvastatin (n = 4808)	Std. Diff
Age group						
18–19	−0.002	0.003	−0.059	−0.002	0.001	−0.033
20–24	−0.002	0.004	−0.040	−0.002	0.003	−0.015
25–29	0.008	0.008	0.004	0.008	0.009	−0.018
30–34	0.012	0.017	−0.039	0.014	0.014	−0.001
35–39	0.029	0.027	0.009	0.031	0.031	−0.001
40–44	0.047	0.057	−0.045	0.050	0.049	0.005
45–49	0.079	0.087	−0.031	0.080	0.083	−0.011
50–54	0.123	0.131	−0.026	0.130	0.133	−0.009
55–59	0.151	0.144	0.020	0.156	0.157	−0.003
60–64	0.165	0.140	0.071	0.159	0.154	0.014
65–69	0.126	0.119	0.023	0.124	0.122	0.008
70–74	0.101	0.103	−0.007	0.098	0.098	−0.002
75–79	0.075	0.084	−0.034	0.070	0.069	0.003
80–84	0.058	0.051	0.029	0.056	0.055	0.004
85–89	0.019	0.021	−0.012	0.018	0.019	−0.007
90–94	0.004	0.004	−0.004	0.004	0.003	0.027
Female	0.485	0.500	−0.030	0.489	0.482	0.014
Disease						
Essential hypertension	0.462	0.457	0.011	0.439	0.444	−0.010
Obesity	0.004	0.003	0.017	0.004	0.003	0.010
CCI	3.055	2.967	0.042	3.061	3.057	0.002
DCSI	0.765	0.729	0.029	0.719	0.735	−0.013
CHA2DS2VASc	2.523	2.534	−0.009	2.491	2.482	0.007
Atherosclerosis of the arteries of the extremities	0.012	0.02	−0.063	0.012	0.013	−0.008
Peripheral arterial occlusive disease	−0.002	−0.001	0.028	−0.002	−0.001	0.038
Peripheral circulatory disorder due to T2D	0.02	0.027	−0.042	0.021	0.02	0.013
Peripheral vascular complication	0.021	0.028	−0.046	0.022	0.021	0.007
Peripheral vascular disease	0.047	0.059	−0.054	0.047	0.048	−0.004
Medications *						
Anti-diabetics	0.002	−0.001	0.041	0.002	−0.001	0.047
ACEI	0.006	0.002	0.063	0.006	0.004	0.027
ARBs	0.006	0.002	0.063	0.006	0.004	0.027
Beta−blockers	0.003	0.003	0.014	0.004	0.002	0.026
Calcium channel blockers	0.005	−0.001	0.084	0.002	−0.001	0.042
Thiazide diuretics	0.009	0.003	0.069	0.007	0.006	0.016
Other diuretics	0.005	0.004	0.015	0.005	0.004	0.015
Nitrates	0.035	0.018	0.110	0.022	0.023	−0.007
Aspirin	0.195	0.224	−0.073	0.176	0.174	0.007
Other antiplatelets	0.011	0.001	0.124	0.004	0.003	0.008
Warfarin	0.009	0.011	−0.016	0.01	0.009	0.003
Digoxin	0.004	0.009	−0.061	0.004	0.005	−0.008
NSAIDs	0.002	0.002	−0.002	0.002	0.001	0.024

* Drugs were grouped by class, and within each class, the drug with the highest standardized difference after PSM was selected to represent the group. Data from other hospitals are listed in Supplementary Materials, Tables S1–S9. Abbreviations: PSM, propensity score matching; CCI, Charlson Comorbidity Index; DCSI, Diabetes Complications Severity Index; Std. diff., standardized difference; ACEIs, angiotensin-converting enzyme inhibitors; ARBs, angiotensin receptor blockers; NSAIDs, nonsteroidal anti-inflammatory drugs.

## Data Availability

The original contributions presented in this study are included in the article and Supplementary Material. Further inquiries can be directed to the corresponding author.

## Supplementary Materials

The following supporting information can be downloaded at: https://www.mdpi.com/article/10.3390/ph18121860/s1, Tables S1–S9: Baseline characteristics before and after propensity score adjustment: Rosuvastatin vs. Atorvastatin (AUMC, GNUH, KDH, KHMC, KHNMC, KWMC, MJH, PNUH, SCHCA); Figure S1: Percentage of patients with recorded LDL-C measurements across different time periods relative to the index date; Figure S2: Percentage distribution of rosuvastatin doses (5–30 mg) as primary (inner circles) and secondary prescribed doses after dose changes (outer circles) among patients within eight hospitals. Percentages reflect only the subset of patients for whom dose information was recorded at each hospital; Figure S3: Percentage distribution of atorvastatin doses (10–80 mg) as primary (inner circles) and secondary prescribed doses after dose changes (outer circles) among patients within ten hospitals. Percentages reflect only the subset of patients for whom dose information was recorded at each hospital; Figure S4: Primary outcomes in preventing cardiovascular events between rosuvastatin and atorvastatin users in the overall population in sensitivity analysis; Figure S5: Secondary outcomes between rosuvastatin and atorvastatin users in the overall population (sensitivity analysis); Figure S6: Safety outcomes between rosuvastatin and atorvastatin users in the overall population (sensitivity analysis); Figure S7: Primary outcomes in preventing cardiovascular events between rosuvastatin and atorvastatin users aged ≥65 years (sensitivity analysis); Figure S8: Safety outcomes between rosuvastatin and atorvastatin users aged ≥65 years (sensitivity analysis).

## Abbreviations

The following abbreviations are used in this manuscript:

AKI	Acute Kidney Injury
AUMC	Ajou University Medical Center
AT	As-Treated
ATMs	Atorvastatin Active Metabolites
CDM	Common Data Model
CVD	Cardiovascular Disease
FeederNet	Federated E-health Big Data for Evidence Renovation Network in Korea
GNUH	Gyeongsang National University Hospital
HDL-C	High-Density Lipoprotein Cholesterol
HR	Hazard Ratio
IRB	Institutional Review Board
ITT	Intention-To-Treat
KDH	Kangdong Sacred Heart Hospital
KHMC	Kyunghee Medical Center
KHNMC	Kyunghee University Hospital at Gangdong
KWMC	Kangwon National University Hospital
LDL-C	Low-Density Lipoprotein Cholesterol
MACE	Major Adverse Cardiovascular Events
MI	Myocardial Infarction
MJH	Myongji Hospital
OMOP-CDM	Observational Medical Outcomes Partnership Common Data Model
OHDSI	Observational Health Data Sciences and Informatics
PAD	Peripheral Arterial Disease
PSM	Propensity Score Matching
PNUH	Pusan National University Hospital
SCHBC	Soonchunhyang University Hospital at Bucheon
SCHCA	Soonchunhyang University Hospital at Cheonan
sdLDL	Small Dense Low-density Lipoprotein
VLDL	Very-Low-Density Lipoprotein
