# Supplementary material for: Rosuvastatin Versus Atorvastatin for Cardiovascular Disease Risk in Patients with Type 2 Diabetes: A Korean Cohort Study
Source: Pharmaceuticals (Basel). 2025 Dec 5;18(12):1860. doi: 10.3390/ph18121860 (PMC12735554; doi:10.3390/ph18121860)
Supplement: Supplementary file 1 [file pharmaceuticals-18-01860-s001.zip › figure legends.pdf]

Supplementary Material figure legends

Figure S1. Percentage of patients with recorded LDL-C measurements across different time periods relative to the index date.

(A) Rosuvastatin, (B) Atorvastatin

LDL-C: Low-density lipoprotein cholesterol

Figure S2. Percentage distribution of rosuvastatin doses (5–30 mg) as primary (inner circles) and secondary prescribed doses after dose changes (outer circles) among patients within eight hospitals. Percentages reflect only the subset of patients for whom dose information was recorded at each hospital.

(A) GNUH, (B) KDH, (C) KHNMC, (D) KWMC, (E) MJH, (F) PNUH, (G) SCHBC, (H) SCHCA

AUMC: Ajou University Medical Center, GNUH: Gyeongsang National University Hospital, KDH: Kangdong Sacred Heart Hospital, KWMC: Kangwon National University Hospital, KHMC: Kyunghee Medical Center, KHNMC: Kyunghee University Hospital at Gangdong, Myongji Hospital, PNUH: Pusan National University Hospital, SCHBC: Soonchunhyang University Hospital at Bucheon, SCHCA: Soonchunhyang University Hospital at Cheonan

Figure S3. Percentage distribution of atorvastatin doses (10–80 mg) as primary (inner circles) and secondary prescribed doses after dose changes (outer circles) among patients within ten hospitals. Percentages reflect only the subset of patients for whom dose information was recorded at each hospital.

(A) AUMC, (B) GNUH, (C) KDH, (D) KHMC, (E) KHNMC, (F) KWMC, (G) MJH, (H) PNUH, (I) SCHBC, (J) SCHCA

AUMC: Ajou University Medical Center, GNUH: Gyeongsang National University Hospital, KDH: Kangdong Sacred Heart Hospital, KWMC: Kangwon National University Hospital,

KHMC: Kyunghee Medical Center, KHNMC: Kyunghee University Hospital at Gangdong, Myongji Hospital, PNUH: Pusan National University Hospital, SCHBC: Soonchunhyang University Hospital at Bucheon, SCHCA: Soonchunhyang University Hospital at Cheonan

Figure S4. Primary outcomes in preventing cardiovascular events between rosuvastatin and atorvastatin users in the overall population in sensitivity analysis

(A) Myocardial infraction, (B) Heart failure, (C) Stroke, (D) Cardiac arrest, (E) In hospital death

AUMC: Ajou University Medical Center, GNUH: Gyeongsang National University Hospital, KDH: Kangdong Sacred Heart Hospital, KWMC: Kangwon National University Hospital, KHMC: Kyunghee Medical Center, KHNMC: Kyunghee University Hospital at Gangdong, Myongji Hospital, PNUH: Pusan National University Hospital, SCHBC: Soonchunhyang University Hospital at Bucheon, SCHCA: Soonchunhyang University Hospital at Cheonan

Outcomes analyzed with fixed-effect models are represented with blue diamonds, while the outcome analyzed with a random-effect model is represented with a gray diamond.

Figure S5. Secondary outcomes between rosuvastatin and atorvastatin users in the overall population (sensitivity analysis)

(A) Peripheral artery disease, (B) Glaucoma

Outcomes analyzed with fixed-effect models are represented with blue diamonds, while the outcome analyzed with a random-effect model is represented with a gray diamond.

AUMC: Ajou University Medical Center, GNUH: Gyeongsang National University Hospital, KDH: Kangdong Sacred Heart Hospital, KWMC: Kangwon National University Hospital, KHMC: Kyunghee Medical Center, KHNMC: Kyunghee University Hospital at Gangdong, Myongji Hospital, PNUH: Pusan National University Hospital, SCHBC: Soonchunhyang University Hospital at Bucheon, SCHCA: Soonchunhyang University Hospital at Cheonan

Figure S6. Safety outcomes between rosuvastatin and atorvastatin users in the overall population (sensitivity analysis)

(A) Acute kidney injury, (B) Cataract, (C) Myalgia

AUMC: Ajou University Medical Center, GNUH: Gyeongsang National University Hospital, KDH: Kangdong Sacred Heart Hospital, KWMC: Kangwon National University Hospital, KHMC: Kyunghee Medical Center, KHNMC: Kyunghee University Hospital at Gangdong, Myongji Hospital, PNUH: Pusan National University Hospital, SCHBC: Soonchunhyang University Hospital at Bucheon, SCHCA: Soonchunhyang University Hospital at Cheonan

Figure S7. Primary outcomes in preventing cardiovascular events between rosuvastatin and atorvastatin users aged ≥ 65 years (sensitivity analysis)

(A) Myocardial infraction, (B) Heart failure, (C) Stroke, (D) Cardiac arrest, (E) In hospital death

Outcomes analyzed with fixed-effect models are represented with blue diamonds, while the outcome analyzed with a random-effect model is represented with a gray diamond.

AUMC: Ajou University Medical Center, GNUH: Gyeongsang National University Hospital, KDH: Kangdong Sacred Heart Hospital, KWMC: Kangwon National University Hospital, KHMC: Kyunghee Medical Center, KHNMC: Kyunghee University Hospital at Gangdong, Myongji Hospital, PNUH: Pusan National University Hospital, SCHBC: Soonchunhyang University Hospital at Bucheon, SCHCA: Soonchunhyang University Hospital at Cheonan

Figure S8. Safety outcomes between rosuvastatin and atorvastatin users aged ≥ 65 years (sensitivity analysis)

(A) Acute kidney injury, (B) Cataract, (C) Myalgia

AUMC: Ajou University Medical Center, GNUH: Gyeongsang National University Hospital, KDH: Kangdong Sacred Heart Hospital, KWMC: Kangwon National University Hospital,

KHMC: Kyunghee Medical Center, KHNMC: Kyunghee University Hospital at Gangdong, Myongji Hospital, PNUH: Pusan National University Hospital, SCHBC: Soonchunhyang University Hospital at Bucheon, SCHCA: Soonchunhyang University Hospital at Cheonan
