# Supplementary material for: Rosuvastatin Versus Atorvastatin for Cardiovascular Disease Risk in Patients with Type 2 Diabetes: A Korean Cohort Study
Source: Pharmaceuticals (Basel). 2025 Dec 5;18(12):1860. doi: 10.3390/ph18121860 (PMC12735554; doi:10.3390/ph18121860)
Supplement: Supplementary file 1 [file pharmaceuticals-18-01860-s001.zip › Table S1.pdf]

Table S1. Baseline characteristics of patients receiving rosuvastatin vs. atorvastatin in the AUMC cohort

	Before PSM adjustment			After PSM adjustment		
	Rosuvastatin (n=2,532)	Atorvastatin (n=5,282)	Std. diff	Rosuvastatin (n=2,187)	Atorvastatin (n=4,808)	Std. diff
Age group						
18-19	-0.001	0.001	-0.015	-0.001	0.001	-0.015
20-24	0.003	0.004	-0.004	0.004	0.003	0.005
25-29	0.008	0.006	0.022	0.009	0.006	0.027
30-34	0.018	0.013	0.043	0.019	0.018	0.009
35-39	0.026	0.027	-0.002	0.027	0.026	0.005
40-44	0.050	0.058	-0.034	0.052	0.051	0.002
45-49	0.093	0.082	0.037	0.094	0.094	0.001
50-54	0.126	0.128	-0.005	0.129	0.132	-0.008
55-59	0.143	0.133	0.029	0.142	0.144	-0.005
60-64	0.146	0.141	0.015	0.146	0.142	0.012
65-69	0.123	0.132	-0.029	0.123	0.123	-0.001
70-74	0.120	0.125	-0.016	0.119	0.119	0.001
75-79	0.079	0.083	-0.015	0.076	0.078	-0.008
80-84	0.045	0.050	-0.022	0.043	0.047	-0.018
85-89	0.016	0.015	0.007	0.014	0.014	0.005
90-94	0.002	0.003	-0.013	0.002	0.002	-0.004
Female	0.455	0.482	-0.053	0.457	0.452	0.010
Disease						
Essential hypertension	0.460	0.452	0.016	0.454	0.448	0.013
Obesity	0.013	0.011	0.020	0.012	0.011	0.016
CCI score	2.673	2.811	-0.060	2.679	2.666	0.006
DCSI	0.398	0.394	0.005	0.385	0.397	-0.015
CHA2DS2VASc	2.450	2.510	-0.049	2.438	2.424	0.011
Atherosclerosis of arteries of the extremities	0.006	0.005	0.005	0.006	0.005	0.01
Peripheral arterial occlusive disease	0.018	0.014	0.031	0.018	0.016	0.012
Peripheral circulatory disorder due to type 2 diabetes mellitus	0.041	0.035	0.03	0.041	0.041	-0.001
Peripheral vascular complication	0.045	0.037	0.039	0.045	0.044	0.004
Peripheral vascular disease	0.062	0.055	0.028	0.061	0.064	-0.01
Medication*						
Anti-diabetic drugs	-0.001	0.002	-0.043	-0.001	0.002	-0.045
ACEI	0.002	0.004	-0.035	0.002	0.004	-0.044
ARBs	0.072	0.097	-0.087	0.073	0.069	0.019
Beta-blockers	-0.001	0.003	-0.041	-0.001	0.003	-0.035
Calcium channel blockers	0.004	0.002	0.026	0.004	0.002	0.026
Thiazide diuretics	0.010	0.007	0.031	0.009	0.008	0.019
Other diuretics	0.002	0.001	0.022	0.002	-0.001	0.035

Nitrates	0.011	0.011	0.001	0.009	0.011	-0.019
Aspirin	0.287	0.250	0.083	0.272	0.282	-0.022
Other antiplatelet drugs	0.009	0.005	0.047	0.008	0.007	0.001
Warfarin	0.013	0.010	0.027	0.013	0.011	0.019
Digoxin	0.008	0.007	0.015	0.008	0.007	0.008
NSAIDs	0.007	0.010	-0.039	0.006	0.008	-0.021

*Drugs were grouped by class, and within each class, only the drug with the highest standardized difference after PSM was selected to represent the group.

PSM, propensity score matching; CCI, Charlson Comorbidity Index; DCSI, Diabetes Complications Severity Index; Std. diff., standardized difference; ACEIs, angiotensin-converting enzyme inhibitors; ARBs, angiotensin receptor blockers; NSAIDs, nonsteroidal anti-inflammatory drugs.
