# Supplementary material for: Rosuvastatin Versus Atorvastatin for Cardiovascular Disease Risk in Patients with Type 2 Diabetes: A Korean Cohort Study
Source: Pharmaceuticals (Basel). 2025 Dec 5;18(12):1860. doi: 10.3390/ph18121860 (PMC12735554; doi:10.3390/ph18121860)
Supplement: Supplementary file 1 [file pharmaceuticals-18-01860-s001.zip › Table S2.pdf]

Table S2. Baseline characteristics of patients receiving rosuvastatin vs. atorvastatin in the GNUH cohort

	Before PSM adjustment			After PSM adjustment		
	Rosuvastatin (n=2,532)	Atorvastatin (n=5,282)	Std. diff	Rosuvastatin (n=2,187)	Atorvastatin (n=4,808)	Std. diff
Age group						
18-19	-0.004	0.003	-0.015	-0.005	0.003	-0.007
20-24	-0.004	0.003	-0.015	-0.004	0.003	-0.004
25-29	-0.004	0.006	-0.059	-0.004	0.005	-0.040
30-34	0.004	0.005	-0.023	0.004	0.005	-0.012
35-39	0.015	0.016	-0.008	0.017	0.015	0.015
40-44	0.023	0.040	-0.098	0.024	0.028	-0.026
45-49	0.046	0.060	-0.061	0.051	0.053	-0.008
50-54	0.082	0.097	-0.052	0.085	0.086	-0.004
55-59	0.141	0.131	0.030	0.148	0.145	0.007
60-64	0.151	0.153	-0.004	0.154	0.149	0.016
65-69	0.162	0.154	0.021	0.160	0.155	0.016
70-74	0.144	0.141	0.008	0.145	0.146	-0.001
75-79	0.128	0.119	0.029	0.125	0.129	-0.013
80-84	0.075	0.050	0.102	0.061	0.056	0.020
85-89	0.023	0.019	0.029	0.019	0.02	-0.006
90-94	-0.004	0.003	-0.022	-0.004	0.003	-0.029
Female	0.466	0.457	0.019	0.469	0.470	-0.003
Disease						
Essential hypertension	0.130	0.122	0.023	0.132	0.127	0.015
Obesity	0.011	0.014	-0.028	0.013	0.014	-0.010
CCI score	2.301	2.397	-0.050	2.319	2.364	-0.024
DCSI	0.799	0.635	0.151	0.770	0.724	0.042
CHA2DS2VASc	2.172	2.092	0.067	2.154	2.132	0.018
Atherosclerosis of arteries of the extremities	0.014	0.015	-0.011	0.013	0.017	-0.038
Peripheral circulatory disorder due to type 2 diabetes mellitus	-0.004	0.006	-0.045	-0.004	0.006	-0.039
Peripheral vascular complication	0.011	0.014	-0.028	0.013	0014	-0.01
Peripheral vascular disease	0.025	0.032	-0.04	0.025	0.033	-0.046
Medication*						
Anti-diabetic drugs	0.004	-0.002	0.083	-0.004	-0.002	0.073
ACEI	0.008	0.014	-0.055	0.008	0.014	-0.062
ARBs	-0.004	0.005	-0.065	-0.004	0.005	-0.056
Beta-blockers	0.044	0.034	0.050	0.034	0.044	-0.054
Calcium channel blockers	-0.004	0.004	-0.073	-0.004	0.004	-0.061
Thiazide diuretics	0.006	0.002	0.065	0.006	0.002	0.064
Other diuretics	0.012	0.007	0.044	0.013	0.007	0.062
Nitrates	0.007	0.009	-0.022	0.006	0.012	-0.060
Aspirin	0.350	0.239	0.246	0.296	0.302	-0.012

Other antiplatelet drugs	0.014	0.002	0.137	0.009	0.003	0.088
Warfarin	0.015	0.017	-0.017	0.016	0.017	-0.006
Digoxin	0.025	0.019	0.038	0.024	0.018	0.042
NSAIDs	0.005	-0.002	0.072	0.006	-0.002	0.075

*Drugs were grouped by class, and within each class, only the drug with the highest standardized difference after PSM was selected to represent the group.

PSM, propensity score matching; CCI, Charlson Comorbidity Index; DCSI, Diabetes Complications Severity Index; Std. diff., standardized difference; ACEIs, angiotensin-converting enzyme inhibitors; ARBs, angiotensin receptor blockers; NSAIDs, nonsteroidal anti-inflammatory drugs.
