# Supplementary material for: Rosuvastatin Versus Atorvastatin for Cardiovascular Disease Risk in Patients with Type 2 Diabetes: A Korean Cohort Study
Source: Pharmaceuticals (Basel). 2025 Dec 5;18(12):1860. doi: 10.3390/ph18121860 (PMC12735554; doi:10.3390/ph18121860)
Supplement: Supplementary file 1 [file pharmaceuticals-18-01860-s001.zip › Table S3.pdf]

Table S3. Baseline characteristics of patients receiving rosuvastatin vs. atorvastatin in the KDH cohort

	Before PSM adjustment			After PSM adjustment		
	Rosuvastatin (n=2,532)	Atorvastatin (n=5,282)	Std. diff	Rosuvastatin (n=2,187)	Atorvastatin (n=4,808)	Std. diff
Age group						
18-19	-0.004	0.003	-0.012	-0.004	0.002	-0.013
20-24	-0.004	0.006	-0.086	-0.004	0.003	-0.047
25-29	0.004	0.007	-0.045	0.004	0.005	-0.022
30-34	0.011	0.011	0.001	0.009	0.011	-0.019
35-39	0.029	0.024	0.031	0.028	0.025	0.019
40-44	0.037	0.040	-0.015	0.036	0.039	-0.014
45-49	0.057	0.065	-0.034	0.058	0.060	-0.007
50-54	0.105	0.104	0.004	0.108	0.104	0.014
55-59	0.131	0.133	-0.005	0.134	0.134	-0.001
60-64	0.148	0.140	0.021	0.150	0.136	0.041
65-69	0.142	0.130	0.034	0.142	0.143	-0.004
70-74	0.111	0.121	-0.032	0.110	0.113	-0.012
75-79	0.107	0.104	0.012	0.107	0.106	0.005
80-84	0.075	0.073	0.009	0.075	0.079	-0.013
85-89	0.034	0.032	0.008	0.029	0.035	-0.031
90-94	0.008	0.007	0.010	0.006	0.007	-0.008
Female	0.493	0.497	-0.007	0.494	0.499	-0.009
Disease						
Essential hypertension	0.166	0.143	0.063	0.165	0.174	-0.026
Obesity	-0.004	0.002	0.002	-0.004	0.002	0.006
CCI score	2.102	2.191	-0.051	2.145	2.160	-0.009
DCSI	0.710	0.793	-0.063	0.724	0.733	-0.007
CHA2DS2VASc	2.142	2.090	0.044	2.146	2.164	-0.015
Atherosclerosis of arteries of the extremities	0.011	0.008	0.032	0.011	0.01	0.012
Peripheral circulatory disorder due to type 2 diabetes mellitus	0.047	0.033	0.069	0.048	0.048	-0.001
Peripheral vascular complication	0.049	0.034	0.074	0.049	0.049	0.003
Medication*						
Anti-diabetic drugs	0.004	0.002	0.025	0.004	0.002	0.032
ACEI	0.004	0.002	0.045	0.005	0.002	0.048
ARBs	0.004	-0.001	0.080	0.004	0.002	0.045
Beta-blockers	-0.004	0.006	-0.089	-0.004	0.003	-0.045
Calcium channel blockers	-0.004	0.004	-0.069	-0.004	0.002	-0.039
Thiazide diuretics	0.109	0.110	-0.005	0.106	0.113	-0.023
Other diuretics	-0.004	0.003	-0.016	-0.004	0.002	-0.020
Nitrates	0.012	0.013	-0.005	0.013	0.011	0.022
Aspirin	0.274	0.337	-0.138	0.278	0.269	0.019
Other antiplatelet drugs	-0.004	0.005	-0.084	-0.004	0.003	-0.053

Warfarin	0.004	0.011	-0.077	0.005	0.007	-0.029
Digoxin	0.005	0.009	-0.042	-0.004	0.005	-0.028
NSAIDs	0.009	0.011	-0.019	0.007	0.010	-0.028

*Drugs were grouped by class, and within each class, only the drug with the highest standardized difference after PSM was selected to represent the group.

PSM, propensity score matching; CCI, Charlson Comorbidity Index; DCSI, Diabetes Complications Severity Index; Std. diff., standardized difference; ACEIs, angiotensin-converting enzyme inhibitors; ARBs, angiotensin receptor blockers; NSAIDs, nonsteroidal anti-inflammatory drugs.
