# Supplementary material for: Rosuvastatin Versus Atorvastatin for Cardiovascular Disease Risk in Patients with Type 2 Diabetes: A Korean Cohort Study
Source: Pharmaceuticals (Basel). 2025 Dec 5;18(12):1860. doi: 10.3390/ph18121860 (PMC12735554; doi:10.3390/ph18121860)
Supplement: Supplementary file 1 [file pharmaceuticals-18-01860-s001.zip › Table S4.pdf]

Table S4. Baseline characteristics of patients receiving rosuvastatin vs. atorvastatin in the KHMC cohort

	Before PSM adjustment			After PSM adjustment		
	Rosuvastatin (n=2,532)	Atorvastatin (n=5,282)	Std. diff	Rosuvastatin (n=2,187)	Atorvastatin (n=4,808)	Std. diff
Age group						
18-19	0.006	0.004	0.029	-0.006	0.006	-0.020
20-24	0.004	0.003	0.023	-0.004	0.002	0.009
25-29	0.005	0.004	0.017	0.006	0.004	0.022
30-34	0.008	0.008	-0.004	0.009	0.009	-0.005
35-39	0.028	0.021	0.046	0.028	0.025	0.017
40-44	0.042	0.032	0.057	0.043	0.038	0.025
45-49	0.068	0.065	0.012	0.069	0.069	0.001
50-54	0.097	0.106	-0.030	0.098	0.102	-0.012
55-59	0.142	0.123	0.057	0.138	0.135	0.009
60-64	0.145	0.163	-0.050	0.148	0.166	-0.050
65-69	0.153	0.148	0.016	0.152	0.146	0.016
70-74	0.129	0.156	-0.076	0.135	0.132	0.008
75-79	0.115	0.098	0.053	0.108	0.105	0.012
80-84	0.044	0.056	-0.052	0.044	0.049	-0.024
85-89	0.016	0.015	0.007	0.016	0.013	0.021
90-94	-0.003	-0.002	0.006	-0.004	-0.002	-0.022
Female	0.532	0.517	0.030	0.530	0.526	0.008
Disease						
Essential hypertension	0.411	0.458	-0.096	0.412	0.399	0.027
Obesity	0.005	0.002	0.057	0.006	0.002	0.053
CCI score	2.991	3.123	-0.063	3.011	3.058	-0.023
DCSI	1.093	0.802	0.252	1.054	1.049	0.004
CHA2DS2VASc	2.723	2.742	-0.016	2.708	2.679	0.026
Atherosclerosis of arteries of the extremities	0.011	0.01	0.008	0.011	0.009	0.018
Peripheral circulatory disorder due to type 2 diabetes mellitus	0.016	0.031	-0.099	0.017	0.018	-0.005
Peripheral vascular disease	0.04	0.05	-0.05	0.043	0.038	0.027
Peripheral vascular disorder due to diabetes mellitus	0.019	0.033	-0.092	0.02	0.02	0.001
Medication*						
Anti-diabetic drugs	0.009	0.010	-0.009	0.007	0.012	-0.060
ACEI	0.035	0.029	0.033	0.034	0.029	0.032
ARBs	0.141	0.091	0.156	0.123	0.132	-0.028
Beta-blockers	-0.003	-0.002	0.018	-0.004	-0.002	0.039
Calcium channel blockers	-0.003	0.010	-0.106	-0.004	0.005	-0.048
Thiazide diuretics	0.079	0.085	-0.023	0.076	0.084	-0.027
Other diuretics	-0.003	0.002	0.016	-0.004	-0.002	0.045
Nitrates	0.056	0.049	0.033	0.049	0.046	0.014

Aspirin	0.226	0.255	-0.067	0.222	0.223	-0.004
Other antiplatelet drugs	-0.003	0.003	-0.027	-0.004	0.003	-0.030
Warfarin	-0.003	0.008	-0.073	-0.004	0.006	-0.051
Digoxin	0.005	0.005	-0.004	0.005	0.004	0.009
NSAIDs	0.005	0.002	0.050	0.006	-0.002	0.069

*Drugs were grouped by class, and within each class, only the drug with the highest standardized difference after PSM was selected to represent the group.

PSM, propensity score matching; CCI, Charlson Comorbidity Index; DCSI, Diabetes Complications Severity Index; Std. diff., standardized difference; ACEIs, angiotensin-converting enzyme inhibitors; ARBs, angiotensin receptor blockers; NSAIDs, nonsteroidal anti-inflammatory drugs.
