# Supplementary material for: Rosuvastatin Versus Atorvastatin for Cardiovascular Disease Risk in Patients with Type 2 Diabetes: A Korean Cohort Study
Source: Pharmaceuticals (Basel). 2025 Dec 5;18(12):1860. doi: 10.3390/ph18121860 (PMC12735554; doi:10.3390/ph18121860)
Supplement: Supplementary file 1 [file pharmaceuticals-18-01860-s001.zip › Table S5.pdf]

Table S5. Baseline characteristics of patients receiving rosuvastatin vs. atorvastatin in the KHNMC cohort

	Before PSM adjustment			After PSM adjustment		
	Rosuvastatin (n=2,532)	Atorvastatin (n=5,282)	Std. diff	Rosuvastatin (n=2,187)	Atorvastatin (n=4,808)	Std. diff
Age group						
18-19	-0.005	-0.002	-0.014	-0.005	-0.002	-0.022
20-24	0.008	0.004	0.059	0.009	0.005	0.042
25-29	0.007	0.006	0.022	0.007	0.006	0.008
30-34	0.013	0.009	0.038	0.013	0.011	0.022
35-39	0.039	0.025	0.079	0.039	0.035	0.019
40-44	0.055	0.052	0.014	0.056	0.058	-0.011
45-49	0.086	0.080	0.022	0.086	0.085	0.003
50-54	0.094	0.116	-0.073	0.098	0.099	-0.004
55-59	0.138	0.151	-0.036	0.142	0.142	0.001
60-64	0.149	0.156	-0.017	0.148	0.160	-0.033
65-69	0.149	0.133	0.046	0.146	0.148	-0.005
70-74	0.122	0.115	0.020	0.119	0.114	0.015
75-79	0.069	0.087	-0.069	0.072	0.073	-0.002
80-84	0.045	0.039	0.033	0.045	0.041	0.018
85-89	0.019	0.021	-0.020	0.017	0.018	-0.008
90-94	0.005	0.004	0.013	-0.005	0.002	0.014
Female	0.480	0.500	-0.040	0.477	0.477	-0.001
Disease						
Essential hypertension	0.388	0.406	-0.037	0.379	0.389	-0.020
Obesity	0.012	0.016	-0.031	0.011	0.017	-0.054
CCI score	2.133	2.094	0.024	2.136	2.093	0.027
DCSI	0.584	0.586	-0.002	0.572	0.569	0.003
CHA2DS2VASc	2.534	2.555	-0.018	2.509	2.500	0.008
Atherosclerosis of arteries of the extremities	-0.005	0.006	-0.035	-0.005	0.004	-0.004
Peripheral circulatory disorder due to type 2 diabetes mellitus	0.028	0.02	0.052	0.028	0.022	0.038
Peripheral vascular disease	0.031	0.023	0.049	0.031	0.026	0.028
Peripheral vascular disorder due to diabetes mellitus	0.028	0.02	0.052	0.028	0.022	0.038
Medication*						
Anti-diabetic drugs	0.009	0.015	-0.050	0.009	0.015	-0.058
ACEI	-0.005	0.007	-0.056	-0.005	0.005	-0.034
ARBs	0.157	0.100	0.170	0.131	0.149	-0.053
Beta-blockers	-0.005	-0.002	0.034	-0.005	-0.002	0.058
Calcium channel blockers	-0.005	0.007	-0.098	-0.005	0.006	-0.082
Thiazide diuretics	0.012	0.004	0.093	0.011	0.006	0.056
Other diuretics	0.010	0.015	-0.041	0.010	0.014	-0.034
Nitrates	0.016	0.014	0.017	0.016	0.011	0.045

Aspirin	0.195	0.196	-0.004	0.190	0.184	0.014
Other antiplatelet drugs	-0.005	-0.002	0.043	-0.005	-0.002	0.053
Warfarin	-0.005	0.009	-0.062	-0.005	0.006	-0.027
Digoxin	-0.005	-0.002	0.034	-0.005	0.003	0.020
NSAIDs	-0.005	0.009	-0.062	-0.005	0.010	-0.084

*Drugs were grouped by class, and within each class, only the drug with the highest standardized difference after PSM was selected to represent the group.

PSM, propensity score matching; CCI, Charlson Comorbidity Index; DCSI, Diabetes Complications Severity Index; Std. diff., standardized difference; ACEIs, angiotensin-converting enzyme inhibitors; ARBs, angiotensin receptor blockers; NSAIDs, nonsteroidal anti-inflammatory drugs.
