# Supplementary material for: Rosuvastatin Versus Atorvastatin for Cardiovascular Disease Risk in Patients with Type 2 Diabetes: A Korean Cohort Study
Source: Pharmaceuticals (Basel). 2025 Dec 5;18(12):1860. doi: 10.3390/ph18121860 (PMC12735554; doi:10.3390/ph18121860)
Supplement: Supplementary file 1 [file pharmaceuticals-18-01860-s001.zip › Table S6.pdf]

Table S6. Baseline characteristics of patients receiving rosuvastatin vs. atorvastatin in the KWMC cohort

	Before PSM adjustment			After PSM adjustment		
	Rosuvastatin (n=2,532)	Atorvastatin (n=5,282)	Std. diff	Rosuvastatin (n=2,187)	Atorvastatin (n=4,808)	Std. diff
Age group						
18-19	-0.002	-0.001	-0.015	-0.003	0.002	-0.035
20-24	0.005	0.002	0.061	0.006	0.003	0.032
25-29	0.003	0.003	0.004	0.004	0.003	0.011
30-34	0.008	0.007	0.018	0.011	0.008	0.025
35-39	0.024	0.019	0.034	0.023	0.025	-0.012
40-44	0.042	0.030	0.062	0.044	0.042	0.010
45-49	0.064	0.046	0.078	0.059	0.060	-0.007
50-54	0.101	0.077	0.082	0.101	0.094	0.023
55-59	0.135	0.111	0.073	0.131	0.134	-0.009
60-64	0.161	0.135	0.075	0.156	0.154	0.004
65-69	0.145	0.149	-0.012	0.142	0.149	-0.020
70-74	0.111	0.148	-0.108	0.115	0.115	-0.001
75-79	0.111	0.135	-0.071	0.112	0.114	-0.008
80-84	0.061	0.093	-0.120	0.067	0.062	0.019
85-89	0.020	0.035	-0.089	0.022	0.024	-0.009
90-94	0.006	0.008	-0.031	0.007	0.009	-0.016
Female	0.497	0.479	0.036	0.488	0.491	-0.006
Disease						
Essential hypertension	0.324	0.219	0.239	0.270	0.271	-0.003
Obesity	-0.002	0.003	-0.014	-0.003	0.004	-0.029
CCI score	2.327	2.123	0.139	2.316	2.298	0.013
DCSI	0.578	0.647	-0.068	0.578	0.554	0.024
CHA2DS2VASc	2.435	2.373	0.050	2.389	2.373	0.013
Atherosclerosis of arteries of the extremities	0.004	0.009	-0.064	0.003	0.005	-0.033
Peripheral arterial occlusive disease	-0.002	0.002	-0.014	-0.003	0.001	0.016
Peripheral circulatory disorder due to type 2 diabetes mellitus	0.012	0.005	0.082	0.006	0.005	0.013
Peripheral vascular complication	0.015	0.018	-0.025	0.009	0.009	0.001
Peripheral vascular disease	0.031	0.037	-0.033	0.027	0.0244	0.016
Medication*						
Anti-diabetic drugs	-0.002	-0.001	0.027	-0.003	-0.001	0.039
ACEI	0.015	0.008	0.069	0.015	0.013	0.023
ARBs	-0.002	-0.001	0.003	-0.003	0.002	-0.035
Beta-blockers	-0.002	-0.001	0.027	-0.003	-0.001	0.041
Calcium channel blockers	0.017	0.017	-0.003	0.019	0.014	0.034
Thiazide diuretics	0.018	0.016	0.019	0.018	0.013	0.036
Other diuretics	0.005	0.015	-0.095	0.005	0.007	-0.026

Nitrates	0.083	0.187	-0.308	0.082	0.076	0.023
Aspirin	0.213	0.378	-0.366	0.234	0.219	0.034
Other antiplatelet drugs	0.070	0.192	-0.368	0.078	0.070	0.030
Warfarin	0.008	0.015	-0.063	0.009	0.008	0.006
Digoxin	0.002	0.014	-0.128	-0.003	0.004	-0.022
NSAIDs	-0.002	-0.001	0.014	-0.003	-0.001	0.045

*Drugs were grouped by class, and within each class, only the drug with the highest standardized difference after PSM was selected to represent the group.

PSM, propensity score matching; CCI, Charlson Comorbidity Index; DCSI, Diabetes Complications Severity Index; Std. diff., standardized difference; ACEIs, angiotensin-converting enzyme inhibitors; ARBs, angiotensin receptor blockers; NSAIDs, nonsteroidal anti-inflammatory drugs.
