# Supplementary material for: Rosuvastatin Versus Atorvastatin for Cardiovascular Disease Risk in Patients with Type 2 Diabetes: A Korean Cohort Study
Source: Pharmaceuticals (Basel). 2025 Dec 5;18(12):1860. doi: 10.3390/ph18121860 (PMC12735554; doi:10.3390/ph18121860)
Supplement: Supplementary file 1 [file pharmaceuticals-18-01860-s001.zip › Table S7.pdf]

**Table S7.** Baseline characteristics of patients receiving rosuvastatin vs. atorvastatin in the MJH cohort

| | Before PSM adjustment | | | After PSM adjustment | | |
|---|---|---|---|---|---|---|
| | Rosuvastatin (n=2,532) | Atorvastatin (n=5,282) | Std. diff | Rosuvastatin (n=2,187) | Atorvastatin (n=4,808) | Std. diff |
| Age group | | | | | | |
| 18-19 | -0.005 | -0.004 | -0.021 | -0.006 | -0.003 | 0.013 |
| 20-24 | -0.004 | 0.002 | -0.012 | -0.005 | -0.002 | 0.001 |
| 25-29 | -0.004 | 0.005 | -0.060 | -0.005 | 0.004 | -0.061 |
| 30-34 | 0.013 | 0.011 | 0.014 | 0.013 | 0.010 | 0.030 |
| 35-39 | 0.020 | 0.029 | -0.053 | 0.022 | 0.025 | -0.017 |
| 40-44 | 0.040 | 0.059 | -0.089 | 0.041 | 0.043 | -0.009 |
| 45-49 | 0.084 | 0.083 | 0.005 | 0.089 | 0.083 | 0.022 |
| 50-54 | 0.110 | 0.115 | -0.017 | 0.114 | 0.109 | 0.014 |
| 55-59 | 0.132 | 0.136 | -0.012 | 0.138 | 0.139 | -0.002 |
| 60-64 | 0.128 | 0.124 | 0.012 | 0.119 | 0.127 | -0.024 |
| 65-69 | 0.120 | 0.112 | 0.025 | 0.121 | 0.120 | 0.005 |
| 70-74 | 0.129 | 0.118 | 0.035 | 0.127 | 0.124 | 0.007 |
| 75-79 | 0.112 | 0.100 | 0.041 | 0.111 | 0.112 | -0.004 |
| 80-84 | 0.075 | 0.067 | 0.030 | 0.071 | 0.073 | -0.006 |
| 85-89 | 0.031 | 0.029 | 0.012 | 0.028 | 0.025 | 0.016 |
| 90-94 | -0.004 | 0.008 | -0.093 | -0.005 | 0.004 | -0.068 |
| Female | 0.504 | 0.486 | 0.036 | 0.501 | 0.497 | 0.008 |
| Disease | | | | | | |
| Essential hypertension | 0.412 | 0.365 | 0.096 | 0.395 | 0.423 | -0.056 |
| Obesity | 0.008 | 0.007 | 0.010 | 0.008 | 0.008 | 0.005 |
| CCI score | 2.144 | 2.253 | -0.069 | 2.164 | 2.147 | 0.011 |
| DCSI | 0.728 | 0.766 | -0.034 | 0.723 | 0.742 | -0.017 |
| CHA2DS2VASc | 2.537 | 2.432 | 0.082 | 2.502 | 2.507 | -0.004 |
| Atherosclerosis of arteries of the extremities | 0.005 | 0.006 | -0.015 | 0.006 | 0.007 | -0.015 |
| Peripheral circulatory disorder due to type 2 diabetes mellitus | 0.008 | -0.002 | 0.1 | 0.006 | 0.002 | 0.063 |
| Peripheral vascular disease | 0.033 | 0.03 | 0.02 | 0.032 | 0.03 | 0.015 |
| Peripheral vascular disorder due to diabetes mellitus | 0.011 | 0.003 | 0.1 | 0.009 | 0.005 | 0.056 |
| Medication* | | | | | | |
| Anti-diabetic drugs | 0.007 | 0.004 | 0.037 | 0.007 | -0.002 | 0.080 |
| ACEI | -0.004 | 0.008 | -0.078 | -0.005 | 0.007 | -0.056 |
| ARBs | -0.004 | 0.008 | -0.089 | -0.005 | 0.005 | -0.058 |
| Beta-blockers | 0.412 | 0.365 | 0.096 | 0.395 | 0.423 | -0.056 |
| Calcium channel blockers | -0.004 | 0.003 | -0.050 | -0.005 | 0.004 | -0.065 |
| Thiazide diuretics | 0.009 | 0.013 | -0.041 | 0.010 | 0.013 | -0.023 |
| Other diuretics | 0.004 | -0.002 | 0.044 | 0.005 | -0.002 | 0.072 |
| Nitrates | 0.091 | 0.043 | 0.191 | 0.056 | 0.070 | -0.060 |

| | | | | | | |
|---|---|---|---|---|---|---|
| Aspirin | 0.307 | 0.226 | 0.184 | 0.278 | 0.299 | -0.047 |
| Other antiplatelet drugs | -0.004 | -0.002 | 0.053 | -0.005 | -0.002 | 0.063 |
| Warfarin | 0.004 | 0.007 | -0.042 | -0.005 | 0.008 | -0.054 |
| Digoxin | 0.015 | 0.011 | 0.036 | 0.014 | 0.012 | 0.019 |
| NSAIDs | 0.006 | 0.004 | 0.027 | 0.006 | 0.004 | 0.037 |

*Drugs were grouped by class, and within each class, only the drug with the highest standardized difference after PSM was selected to represent the group.

PSM, propensity score matching; CCI, Charlson Comorbidity Index; DCSI, Diabetes Complications Severity Index; Std. diff., standardized difference; ACEIs, angiotensin-converting enzyme inhibitors; ARBs, angiotensin receptor blockers; NSAIDs, nonsteroidal anti-inflammatory drugs.
