# Supplementary material for: Rosuvastatin Versus Atorvastatin for Cardiovascular Disease Risk in Patients with Type 2 Diabetes: A Korean Cohort Study
Source: Pharmaceuticals (Basel). 2025 Dec 5;18(12):1860. doi: 10.3390/ph18121860 (PMC12735554; doi:10.3390/ph18121860)
Supplement: Supplementary file 1 [file pharmaceuticals-18-01860-s001.zip › Table S8.pdf]

Table S8. Baseline characteristics of patients receiving rosuvastatin vs. atorvastatin in the PNUH cohort

	Before PSM adjustment			After PSM adjustment		
	Rosuvastatin (n=2,532)	Atorvastatin (n=5,282)	Std. diff	Rosuvastatin (n=2,187)	Atorvastatin (n=4,808)	Std. diff
Age group						
18-19	-0.005	-0.003	-0.002	-0.006	-0.003	0.006
20-24	-0.005	-0.003	-0.002	-0.006	-0.003	0.006
25-29	0.007	0.004	0.037	0.008	0.005	0.041
30-34	-0.005	0.008	-0.047	-0.006	0.009	-0.052
35-39	0.015	0.016	-0.013	0.015	0.016	-0.007
40-44	0.032	0.030	0.010	0.033	0.029	0.020
45-49	0.062	0.052	0.045	0.062	0.048	0.058
50-54	0.076	0.074	0.008	0.076	0.073	0.009
55-59	0.116	0.129	-0.040	0.118	0.129	-0.036
60-64	0.151	0.147	0.012	0.152	0.152	-0.001
65-69	0.173	0.181	-0.021	0.177	0.177	-0.001
70-74	0.170	0.166	0.011	0.168	0.169	-0.001
75-79	0.123	0.111	0.038	0.122	0.114	0.027
80-84	0.058	0.062	-0.017	0.051	0.060	-0.042
85-89	0.011	0.017	-0.055	0.011	0.013	-0.021
90-94	-0.005	-0.003	-0.029	-0.006	-0.003	-0.027
Female	0.464	0.497	-0.065	0.466	0.454	0.024
Disease						
Essential hypertension	0.485	0.450	0.071	0.474	0.490	-0.032
Obesity	0.007	0.004	0.037	-0.006	0.006	-0.016
CCI score	3.420	3.506	-0.033	3.402	3.519	-0.045
DCSI	1.089	1.050	0.029	1.081	1.059	0.017
CHA2DS2VASc	2.745	2.755	-0.009	2.717	2.745	-0.023
Atherosclerosis of arteries of the extremities	-0.005	0.011	-0.091	-0.006	0.013	-0.105
Peripheral circulatory disorder associated with type 2 diabetes mellitus	0.02	0.016	0.032	0.018	0.015	0.023
Peripheral vascular disease	0.019	0.027	-0.057	0.017	0.028	-0.077
Peripheral vascular disorder due to diabetes mellitus	0.028	0.025	0.023	0.026	0.024	0.015
Medication*						
Anti-diabetic drugs	0.013	0.006	0.067	0.012	0.004	0.088
ACEI	-0.005	-0.003	0.06	-0.006	-0.003	0.069
ARBs	0.053	0.042	0.049	0.051	0.039	0.054
Beta-blockers	0.008	0.003	0.066	0.009	-0.003	0.089
Calcium channel blockers	-0.005	0.011	-0.096	-0.006	0.010	-0.102
Thiazide diuretics	0.013	-0.003	0.121	0.012	-0.003	0.115
Other diuretics	0.025	0.040	-0.083	0.023	0.032	-0.056
Nitrates	0.011	0.006	0.055	0.011	0.005	0.073

Aspirin	0.257	0.256	0.002	0.257	0.274	-0.037
Other antiplatelet drugs	0.006	0.010	-0.037	0.006	0.010	-0.052
Warfarin	0.018	0.014	0.031	0.018	0.012	0.042
Digoxin	0.011	0.011	-0.007	0.011	0.013	-0.014
NSAIDs	0.016	0.008	0.073	0.017	0.008	0.073

*Drugs were grouped by class, and within each class, only the drug with the highest standardized difference after PSM was selected to represent the group.

PSM, propensity score matching; CCI, Charlson Comorbidity Index; DCSI, Diabetes Complications Severity Index; Std. diff., standardized difference; ACEIs, angiotensin-converting enzyme inhibitors; ARBs, angiotensin receptor blockers; NSAIDs, nonsteroidal anti-inflammatory drugs.
