# Supplementary material for: Rosuvastatin Versus Atorvastatin for Cardiovascular Disease Risk in Patients with Type 2 Diabetes: A Korean Cohort Study
Source: Pharmaceuticals (Basel). 2025 Dec 5;18(12):1860. doi: 10.3390/ph18121860 (PMC12735554; doi:10.3390/ph18121860)
Supplement: Supplementary file 1 [file pharmaceuticals-18-01860-s001.zip › Table S9.pdf]

Table S9. Baseline characteristics of patients receiving rosuvastatin vs. atorvastatin in the SCHCA cohort

	Before PSM adjustment			After PSM adjustment		
	Rosuvastatin (n=2,532)	Atorvastatin (n=5,282)	Std. diff	Rosuvastatin (n=2,187)	Atorvastatin (n=4,808)	Std. diff
Age group						
18-19	-0.003	0.001	-0.011	-0.003	-0.001	-0.024
20-24	0.004	0.005	-0.019	0.004	0.005	-0.010
25-29	0.007	0.008	-0.012	0.008	0.008	-0.006
30-34	0.013	0.018	-0.039	0.014	0.018	-0.027
35-39	0.026	0.031	-0.032	0.027	0.029	-0.013
40-44	0.045	0.056	-0.053	0.046	0.044	0.010
45-49	0.054	0.075	-0.086	0.056	0.052	0.015
50-54	0.108	0.114	-0.018	0.110	0.104	0.018
55-59	0.149	0.137	0.034	0.150	0.130	0.057
60-64	0.149	0.139	0.029	0.145	0.149	-0.012
65-69	0.133	0.117	0.049	0.131	0.134	-0.011
70-74	0.132	0.121	0.033	0.131	0.132	-0.005
75-79	0.108	0.103	0.016	0.108	0.115	-0.022
80-84	0.053	0.053	0.002	0.053	0.058	-0.023
85-89	0.016	0.019	-0.018	0.015	0.02	-0.037
90-94	-0.003	0.003	-0.028	-0.003	0.003	-0.029
Female	0.472	0.470	0.003	0.475	0.467	0.014
Disease						
Essential hypertension	0.510	0.464	0.093	0.497	0.511	-0.027
Obesity	0.011	0.007	0.044	0.011	0.009	0.023
CCI score	2.874	2.784	0.041	2.849	2.864	-0.007
DCSI	0.901	0.727	0.146	0.869	0.885	-0.013
CHA2DS2VASc	2.709	2.620	0.077	2.692	2.729	-0.031
Atherosclerosis of arteries of the extremities	0.033	0.017	0.106	0.029	0.026	0.021
Peripheral circulatory disorder due to type 2 diabetes mellitus	0.024	0.034	-0.063	0.024	0.025	-0.003
Peripheral vascular complication	0.025	0.037	-0.067	0.026	0.027	-0.008
Peripheral vascular disease	0.065	0.058	0.027	0.061	0.06	0.006
Medication*						
Anti-diabetic drugs	0.575	0.629	-0.109	0.585	0.555	0.061
ACEI	0.013	0.006	0.075	0.013	0.008	0.047
ARBs	0.008	0.005	0.038	0.009	0.006	0.035
Beta-blockers	-0.003	0.003	-0.021	-0.003	0.003	-0.046
Calcium channel blockers	0.003	-0.001	0.050	0.003	-0.001	0.040
Thiazide diuretics	0.099	0.108	-0.028	0.101	0.095	0.020
Other diuretics	-0.003	0.002	-0.036	-0.003	0.002	-0.045
Nitrates	0.081	0.043	0.156	0.067	0.073	-0.023
Aspirin	0.299	0.287	0.027	0.296	0.302	-0.013

Other antiplatelet drugs	0.207	0.144	0.168	0.194	0.209	-0.038
Warfarin	0.005	0.004	0.013	0.006	0.005	0.010
Digoxin	0.004	0.004	-0.015	0.004	0.005	-0.022
NSAIDs	0.004	-0.001	0.075	0.004	-0.001	0.079

*Drugs were grouped by class, and within each class, only the drug with the highest standardized difference after PSM was selected to represent the group.

PSM, propensity score matching; CCI, Charlson Comorbidity Index; DCSI, Diabetes Complications Severity Index; Std. diff., standardized difference; ACEIs, angiotensin-converting enzyme inhibitors; ARBs, angiotensin receptor blockers; NSAIDs, nonsteroidal anti-inflammatory drugs.
