# Supplementary figures and images for: Rosuvastatin Versus Atorvastatin for Cardiovascular Disease Risk in Patients with Type 2 Diabetes: A Korean Cohort Study
Source: Pharmaceuticals (Basel). 2025 Dec 5;18(12):1860. doi: 10.3390/ph18121860 (PMC12735554; doi:10.3390/ph18121860)

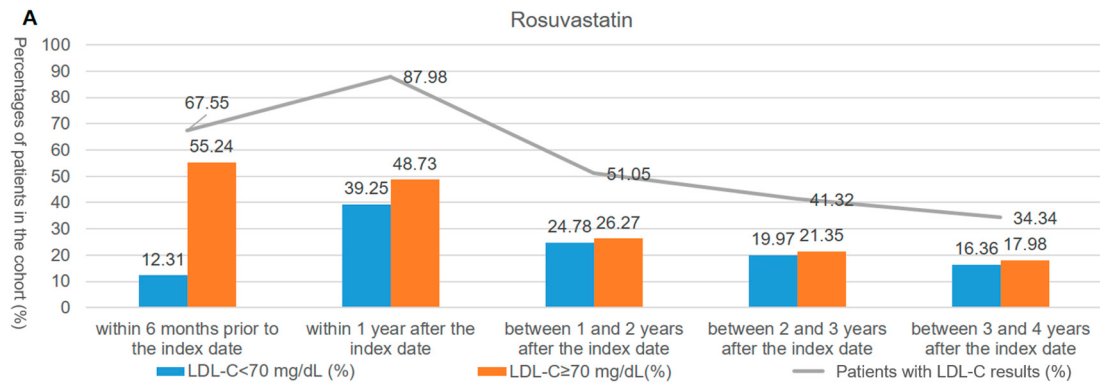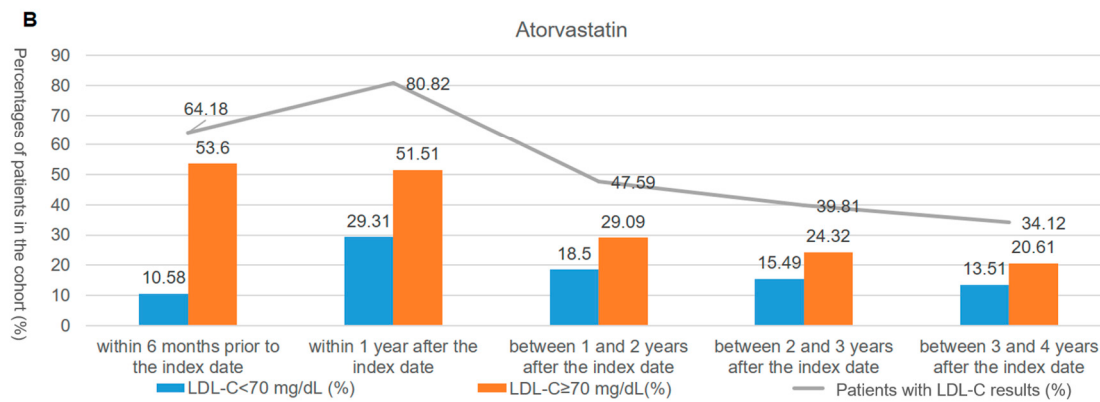

Supplement: Supplementary file 1 [file pharmaceuticals-18-01860-s001.zip › Figure S1.pdf]

Rosuvastatin

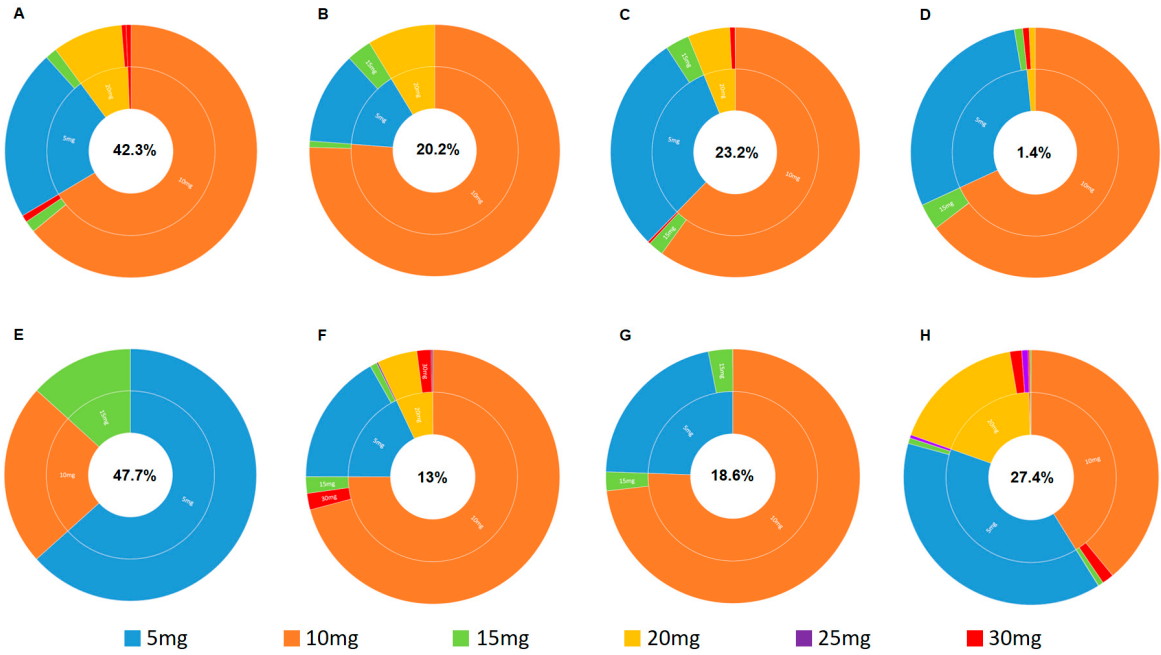

Supplement: Supplementary file 1 [file pharmaceuticals-18-01860-s001.zip › Figure S2.pdf]

Atorvastatin

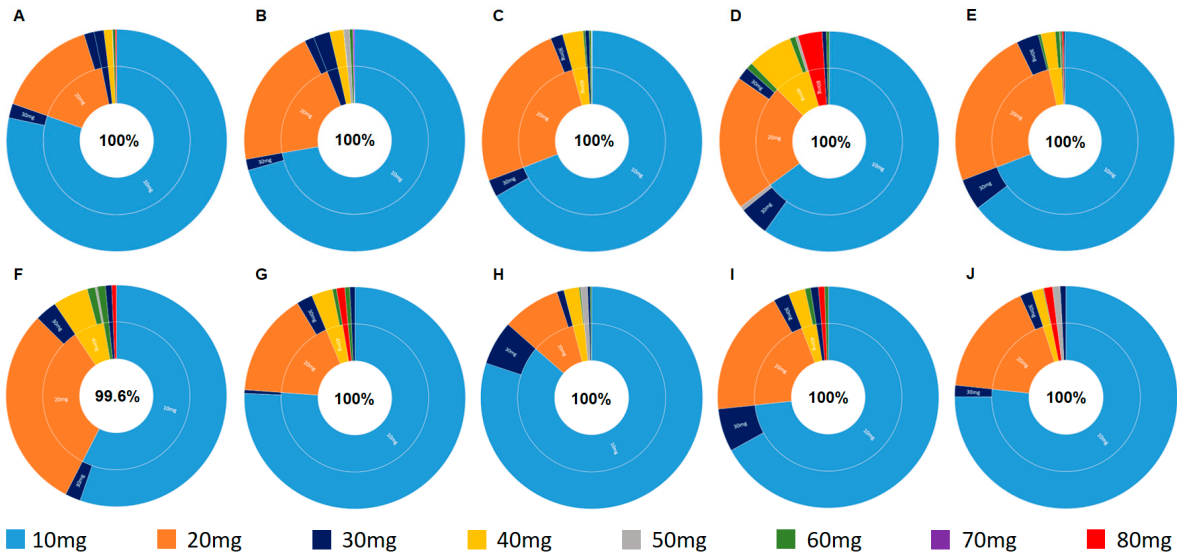

Supplement: Supplementary file 1 [file pharmaceuticals-18-01860-s001.zip › Figure S3.pdf]

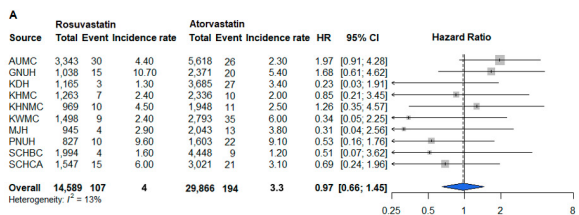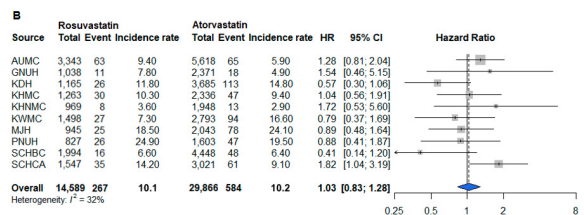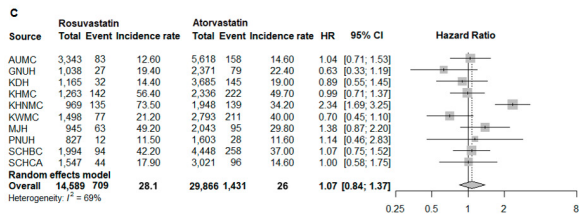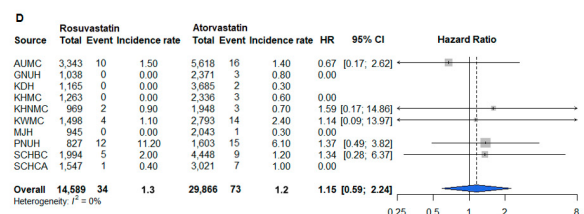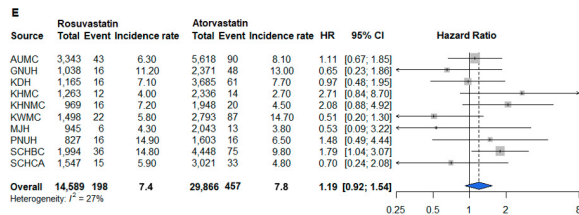

Supplement: Supplementary file 1 [file pharmaceuticals-18-01860-s001.zip › Figure S4.pdf]

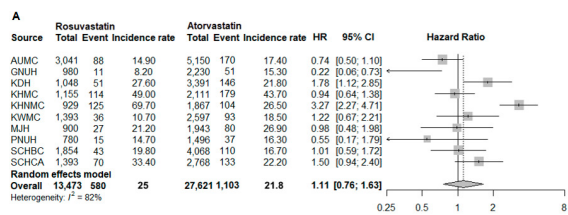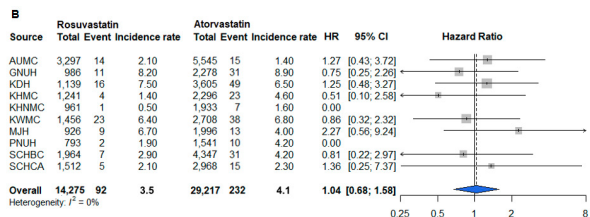

Supplement: Supplementary file 1 [file pharmaceuticals-18-01860-s001.zip › Figure S5.pdf]

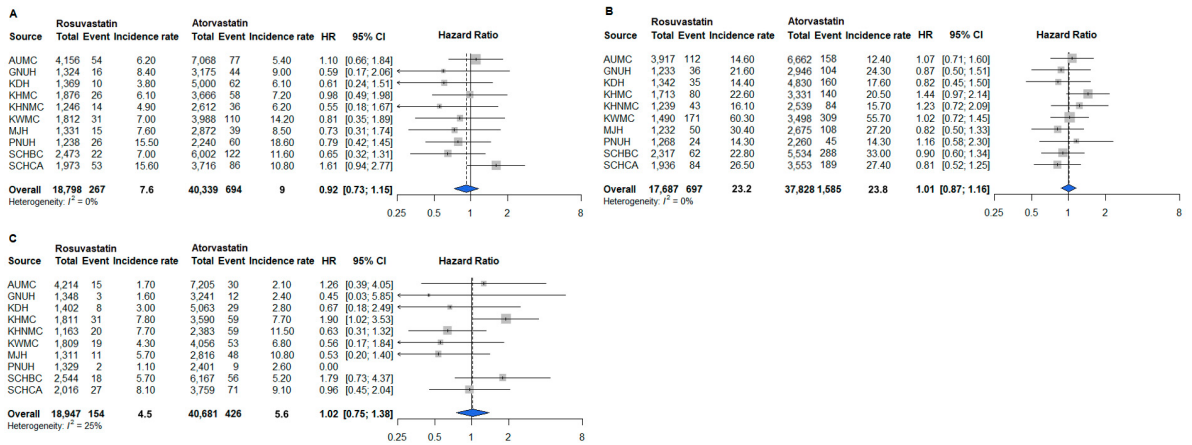

Supplement: Supplementary file 1 [file pharmaceuticals-18-01860-s001.zip › Figure S6.pdf]

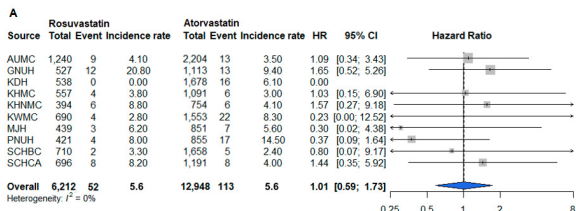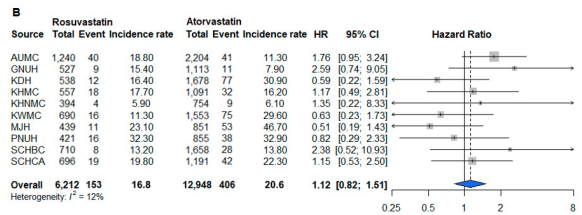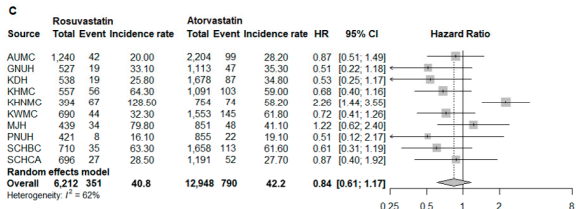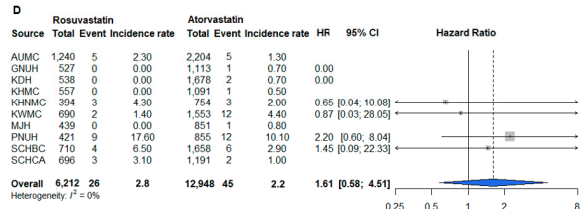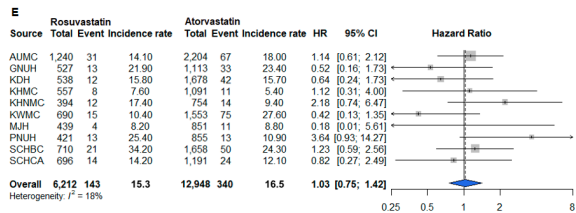

Supplement: Supplementary file 1 [file pharmaceuticals-18-01860-s001.zip › Figure S7.pdf]

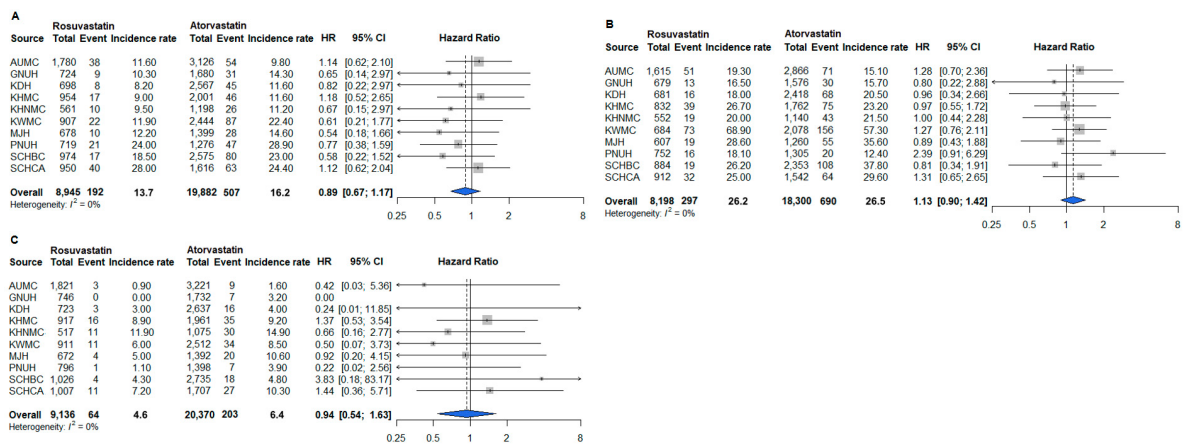

Supplement: Supplementary file 1 [file pharmaceuticals-18-01860-s001.zip › Figure S8.pdf]
